# Inflammatory biomarkers for neurobehavioral dysregulation in former American football players: findings from the DIAGNOSE CTE Research Project

**DOI:** 10.1186/s12974-024-03034-6

**Published:** 2024-02-09

**Authors:** Suzan van Amerongen, Surya V. Pulukuri, Fatima Tuz-Zahra, Yorghos Tripodis, Jonathan D. Cherry, Charles Bernick, Yonas E. Geda, Jennifer V. Wethe, Douglas I. Katz, Michael L. Alosco, Charles H. Adler, Laura J. Balcer, Nicholas J. Ashton, Kaj Blennow, Henrik Zetterberg, Daniel H. Daneshvar, Elizabeth A. Colasurdo, Jeffrey J. Iliff, Gail Li, Elaine R. Peskind, Martha E. Shenton, Eric M. Reiman, Jeffrey L. Cummings, Robert A. Stern, for the DIAGNOSE CTE Research Project Investigators, Kewei Chen, Hillary Protas, Eric Reiman, Yi Su, Connie Boker, Michael L. Alosco, Rhoda Au, Robert C. Cantu, Lindsay Farrer, Robert Helm, Douglas I. Katz, Neil Kowall, Jesse Mez, Gustavo Mercier, James Otis, Robert A. Stern, Jason Weller, Tahlia Bragg, Irene Simkin, Diana Trujillo-Rodriguez, Suzan van Amerongen, Alondra Andino, Shannon Conneely, Courtney Diamond, Tessa Fagle, Olivia Haller, Tennyson Hunt, Nicole Gullotti, Bailey Kossow, Carrie Kugelmass, Megan Mariani, Brian Mayville, Kathleen McLaughlin, Mary Nanna, Marty DiPopolo, Taylor Platt, Surya Pulukuri, Fiona Rice, Madison Sestak, Irene Simkin, Michael McClean, Yorghos Tripodis, Douglas Annis, Christine Chaisson, Diane B. Dixon, Carolyn Finney, Kerrin Gallagher, Kaitlin Hartlage, Jun Lu, Brett Martin, Emmanuel Ojo, Joseph N. Palmisano, Brittany Pine, Janani Ramachandran, Zachary Baucom, Fatima Tuz-Zahra, Eukyung Yhang, Sylvain Bouix, Jennifer Fitzsimmons, Alexander P. Lin, Inga K. Koerte, Ofer Pasternak, Martha E. Shenton, Hector Arciniega, Tashrif Billah, Elena Bonke, Katherine Breedlove, Holly Carrington, Eduardo Coello, Michael J. Coleman, Omar John, Leonard Jung, Huijun Liao, Maria Loy, Elizabeth Rizzoni, Vivian Schultz, Annelise Silva, Brynn Vessey, Tim L. T. Wiegand, Sarah Banks, Charles Bernick, Jason Miller, Aaron Ritter, Marwan Sabbagh, Raelynn de la Cruz, Jan Durant, Morgan Golceker, Nicolette Harmon, Jaeson Kaylegian, Rachelle Long, Christin Nance, Priscilla Sandoval, Miranda Staples, Robert W. Turner, Emma F. Clark, Kenneth L. Marek, Andrew Serrano, Charles H. Adler, David W. Dodick, Yonas Geda, Jennifer V. Wethe, Amy Duffy, Bryce Falk, Marci Howard, Michelle Montague, Thomas Osgood, Debra Babcock, Patrick Bellgowan, Laura Balcer, William Barr, Judith Goldberg, Binu Joseph, Ivan Kirov, Yvonne Lui, Charles Marmar, Thomas Wisniewski, Alhassan Al-Kharafi, Allan George, Lisena Hasanaj, Sammie Martin, Edward Riley, William Runge, Liliana Serrano, Nicholas Ashton, Henrik Zetterberg, Kaj Blennow, Jeffrey L. Cummings, Jeffrey Iliff, Gail Li, Deidre Janssen, James Meabon, Elaine R. Peskind, Juan Piantino, Abigail Schindler, Ronald Thomas, Elizabeth Colasurdo, Jane Shofer, Daniel S. Marcus, Jenny Gurney, Richard Greenwald, Keith A. Johnson

**Affiliations:** 1 Boston University CTE Center, Boston University Chobanian & Avedisian School of Medicine, Boston, MA USA; 2 Alzheimer Center Amsterdam, Neurology, Vrije Universiteit Amsterdam, Amsterdam UMC Location VUmc, Amsterdam, The Netherlands; 3 Amsterdam Neuroscience, Neurodegeneration, Amsterdam, The Netherlands; 4 Department of Biostatistics, Boston University School of Public Health, Boston, MA USA; 5 Boston University Alzheimer’s Disease Research Center, Boston University Chobanian & Avedisian School of Medicine, Boston, MA USA; 6 VA Boston Healthcare System, U.S. Department of Veteran Affairs, Boston, MA USA; 7 Department of Veterans Affairs Medical Center, Bedford, MA USA; 8 Department of Pathology and Laboratory Medicine, Boston University Chobanian & Avedisian School of Medicine, Boston, MA USA; 9 Cleveland Clinic Lou Ruvo Center for Brain Health, Las Vegas, NV USA; 10 Department of Neurology and the Franke Global Neuroscience Education Center, Barrow Neurological Institute, Phoenix, AZ USA; 11 Department of Psychiatry and Psychology, Mayo Clinic School of Medicine, Mayo Clinic Arizona, Scottsdale, AZ USA; 12 Department of Neurology, Boston University Chobanian & Avedisian School of Medicine, Boston, MA USA; 13 Brain Injury Program, Encompass Health Braintree Rehabilitation Hospital, Braintree, MA USA; 14 Department of Neurology, Mayo Clinic College of Medicine, Mayo Clinic Arizona, Scottsdale, AZ USA; 15 Departments of Neurology, Population Health and Ophthalmology, NYU Grossman School of Medicine, New York, NY USA; 16 Department of Psychiatry and Neurochemistry, Institute of Neuroscience and Physiology, The Sahlgrenska Academy at the University of Gothenburg, Mölndal, Sweden; 17 Clinical Neurochemistry Laboratory, Sahlgrenska University Hospital, Mölndal, Sweden; 18 Institute of Psychiatry, Psychology and Neuroscience, King’s College London, Maurice Wohl Institute Clinical Neuroscience Institute, London, UK; 19 Department of Neurodegenerative Disease, UCL Institute of Neurology, Queen Square, London, UK; 20 UK Dementia Research Institute at UCL, London, UK; 21 Hong Kong Center for Neurodegenerative Diseases, Clear Water Bay, Hong Kong, China; 22 Wisconsin Alzheimer’s Disease Research Center, University of Wisconsin School of Medicine and Public Health, University of Wisconsin-Madison, Madison, WI 53792 USA; 23 Department of Physical Medicine and Rehabilitation, Harvard Medical School, Boston, MA USA; 24 Veterans Affairs Northwest Mental Illness Research, Education, and Clinical Center, Seattle, WA USA; 25 Department of Psychiatry and Behavioral Sciences, University of Washington School of Medicine, Seattle, WA USA; 26 Education, and Clinical Center, Veterans Affairs Puget Sound Health Care System Geriatric Research, Seattle, WA USA; 27 Psychiatry Neuroimaging Laboratory, Harvard Medical School, Departments of Psychiatry and Radiology, Brigham and Women’s Hospital, Boston, MA USA; 28 Banner Alzheimer’s Institute, University of Arizona, Arizona State University, Translational Genomics Research Institute, and Arizona Alzheimer’s Consortium, Phoenix, AZ USA; 29 Chambers-Grundy Center for Transformative Neuroscience, Department of Brain Health, School of Integrated Health Sciences, University of Nevada Las Vegas, Las Vegas, NV USA; 30 Departments of Neurosurgery, and Anatomy and Neurobiology, Boston University Chobanian & Avedisian School of Medicine, Boston, MA USA

**Keywords:** Repetitive head impacts, Chronic traumatic encephalopathy, Traumatic encephalopathy syndrome, Neurodegeneration, Neuroinflammation, Neuropsychiatric symptoms, Biomarkers, CSF, IL-6, Neurofilament light chain protein

## Abstract

**Background:**

Traumatic encephalopathy syndrome (TES) is defined as the clinical manifestation of the neuropathological entity chronic traumatic encephalopathy (CTE). A core feature of TES is neurobehavioral dysregulation (NBD), a neuropsychiatric syndrome in repetitive head impact (RHI)-exposed individuals, characterized by a poor regulation of emotions/behavior. To discover biological correlates for NBD, we investigated the association between biomarkers of inflammation (interleukin (IL)-1β, IL-6, IL-8, IL-10, C-reactive protein (CRP), tumor necrosis factor (TNF)-α) in cerebrospinal fluid (CSF) and NBD symptoms in former American football players and unexposed individuals.

**Methods:**

Our cohort consisted of former American football players, with (*n* = 104) or without (*n* = 76) NBD diagnosis, as well as asymptomatic unexposed individuals (*n* = 55) from the DIAGNOSE CTE Research Project. Specific measures for NBD were derived (i.e., explosivity, emotional dyscontrol, impulsivity, affective lability, and a total NBD score) from a factor analysis of multiple self-report neuropsychiatric measures. Analyses of covariance tested differences in biomarker concentrations between the three groups. Within former football players, multivariable linear regression models assessed relationships among log-transformed inflammatory biomarkers, proxies for RHI exposure (total years of football, cumulative head impact index), and NBD factor scores, adjusted for relevant confounding variables. Sensitivity analyses tested (1) differences in age subgroups (< 60, ≥ 60 years); (2) whether associations could be identified with plasma inflammatory biomarkers; (3) associations between neurodegeneration and NBD, using plasma neurofilament light (NfL) chain protein; and (4) associations between biomarkers and cognitive performance to explore broader clinical symptoms related to TES.

**Results:**

CSF IL-6 was higher in former American football players with NBD diagnosis compared to players without NBD. Furthermore, elevated levels of CSF IL-6 were significantly associated with higher emotional dyscontrol, affective lability, impulsivity, and total NBD scores. In older football players, plasma NfL was associated with higher emotional dyscontrol and impulsivity, but also with worse executive function and processing speed. Proxies for RHI exposure were not significantly associated with biomarker concentrations.

**Conclusion:**

Specific NBD symptoms in former American football players may result from multiple factors, including neuroinflammation and neurodegeneration. Future studies need to unravel the exact link between NBD and RHI exposure, including the role of other pathophysiological pathways.

**Supplementary Information:**

The online version contains supplementary material available at 10.1186/s12974-024-03034-6.

## Introduction

Concerns have been raised regarding the long-term sequelae of repetitive concussive and subconcussive impacts on the brain, particularly among athletes engaged in contact sports such as American football. Exposure to these repetitive head impacts (RHI) is linked to a unique neurodegenerative disease, known as chronic traumatic encephalopathy (CTE) [1]. Although the precise clinical manifestation of CTE remains elusive, an expert panel developed the 2021 National Institute of Neurological Disorders and Stroke (NINDS) Consensus Diagnostic Criteria for Traumatic Encephalopathy Syndrome (TES), defined as the clinical syndrome underlying CTE [2]. TES consists of three core clinical features, of which two are cognitive impairment and neurobehavioral dysregulation (NBD). The latter describes a neuropsychiatric syndrome that includes features of explosiveness, having a short fuse, impulsivity, and emotional lability, collectively representing a poor regulation of emotions and behaviors [2].

The pathogenesis underlying NBD in former contact-sport athletes is yet unclear. The deposition of peri-vascular phosphorylated tau (p-tau) in the sulcal depths is the main feature of CTE pathology [3, 4]. Experimental and pathological studies have also demonstrated an association between RHI exposure and chronic neuroinflammation, which may mediate the development of neuronal degeneration and CTE p-tau pathology [4–8]. Neuroinflammation is closely linked to other neurodegenerative diseases and contributes to their pathophysiology and progression [9]. Key features of neuroinflammation include microglial activation and the release of inflammatory cytokines. These cytokines in cerebrospinal fluid (CSF) have therefore been suggested as relevant biomarkers for neuroinflammation. Elevated concentrations of pro-inflammatory cytokines in CSF have been observed in various neurological conditions, including Alzheimer’s disease (AD) [10] and severe traumatic brain injury (TBI) [11]. Pro-inflammatory cytokines have been associated with neuropsychiatric symptoms such as aggression [12–14], depressive symptoms [13], disinhibition [15], and apathy [13] in these neurological disorders. It is possible that neuroinflammation is involved in the development or progression of NBD in RHI-exposed athletes, but no studies have investigated this to date. A better understanding of this relationship may provide insights into the pathogenesis of NBD in individuals with RHI exposure and TES.

Here, we study the relationship between inflammatory biomarkers in CSF, NBD symptoms and exposure to RHI. This CSF panel includes interleukin (IL)-1β, IL-6, IL-8, IL-10, C-reactive protein (CRP), and tumor necrosis factor-alpha (TNF-α); inflammatory cytokines that have been linked to neuropsychiatric symptoms occurring in neurological and psychiatric disorders. We compare concentrations of inflammatory biomarkers between former American football players with and without NBD, as well as a cohort of asymptomatic unexposed healthy individuals. Within all former football players, we examine the association of CSF inflammatory biomarkers with specific NBD measures and estimates of RHI exposure. As sensitivity analyses, we test the effects in age subgroups (< 60, ≥ 60 years), re-examine associations with the same biomarkers but in plasma, assess the association between neurodegeneration and NBD using plasma neurofilament light (NfL) chain protein, and the association between biomarkers and cognitive performance, to explore broader clinical symptoms related to TES.

## Methods

### Study design and cohort

This study is part of the Diagnostics, Imaging, and Genetics Network for the Objective Study and Evaluation of Chronic Traumatic Encephalopathy (DIAGNOSE CTE) Research Project, an 8-year multicenter prospective study aiming to identify biomarkers for detecting CTE during life and characterize its clinical features [16]. The project enrolled 240 male individuals aged 45–74 years old, including former professional American football players (*n* = 120), former college American football players (*n* = 60), and asymptomatic individuals without a history of traumatic brain injury or RHI (*n* = 60). We conducted baseline visits between September 2016 and February 2020. Institutional Review Boards from each study site approved the study and participants gave written informed consent prior to any study procedures. Additional detailed information about the methods and design of the DIAGNOSE Research Project is reported elsewhere [16]. After retrospectively uncovering significant medical information, four unexposed individuals were excluded from subsequent analyses, as their revealed details impacted the presumed 'asymptomatic/healthy' nature of this cohort. This led to a cohort of 180 former American football players and 56 unexposed individuals.

### Neurobehavioral dysregulation

During the DIAGNOSE CTE Multidisciplinary Diagnostic Consensus Conferences, a multidisciplinary panel provided a research TES diagnosis to each participant, including a diagnosis of neurobehavioral dysregulation, guided by the NINDS Consensus Diagnostic Criteria for TES [2, 16]. There were 104 former football players and one unexposed individual that received a research NBD diagnosis. Based on their NBD diagnosis, three groups were created: (1) former football players with NBD (*n* = 104); (2) former football players without NBD (*n* = 76); (3) unexposed individuals without NBD (*n* = 55, one unexposed individual with NBD was excluded [17–19]).

Furthermore, specific measures for NBD were developed using a stepwise approach, as explained in detail elsewhere [17] and summarized in Additional file 1. Expert-driven confirmatory factor analyses with items from relevant self-reported neuropsychiatric rating scales were used to generate four NBD factor scores: explosivity, emotional dyscontrol, affective lability (reflecting sudden tendencies to cry), impulsivity, and a total NBD score.

### Inflammatory biomarkers in CSF

As part of the DIAGNOSE CTE Research Project, CSF was collected via lumbar puncture, as described elsewhere [16]. CSF samples were measured for IL-1β, IL-6, IL-8, IL-10, and TNF-α with the V-PLEX Proinflammatory and Vascular Injury Panels (Meso Scale Diagnostics LLC, Rockville, Maryland). Further specifics are summarized in Additional file 1. CSF biomarkers were available for 137 former football players (76 with NBD diagnosis, 61 without NBD diagnosis) and 41 unexposed individuals.

### Exposure to repetitive head impacts

Football history was obtained through semi-structured interviews, including the duration of play at each level (youth, high school, college, professional) and the position at the highest level of play (e.g., college or professional). Total years of football across all levels of play were used as the primary measure of exposure. The cumulative head impact index (CHII) was used as a secondary measure of exposure. The CHII is an exposure metric that estimates the total load of linear and rotational head impacts during a football career. It combines history-based athletic information and estimates from helmet accelerometer studies. The development of this metric is described previously [18]. For our analyses, we focused on the CHII measure for linear acceleration, expressed in g-force (*g*).

### Sample features

Demographic characteristics, medical history, medication use, and alcohol/substance use were collected through interviews and questionnaires. Race and ethnicity were self-identified, participants self-identified as ‘White’, ‘Black or African American’, ‘Native Hawaiian or other Pacific Islanders’ and ‘Multiple Races’. For analysis purposes, we combined the latter three into one variable, resulting in two race categories. Body mass index (BMI) was calculated using current height and weight. The Alcohol Use Disorders Identification Test (AUDIT), a 10-item screening tool, provided a total score for alcohol consumption (range 0–40) [19]. Apolipoprotein E (*APOE*) genotyping was performed to identify carriers and non-carriers of the *APOE-ε4* allele. The revised Framingham Stroke Risk Profile (rFSRP) score was calculated to determine vascular risk factors. [20] Other relevant medical history included a self-reported questionnaire on sleep apnea diagnosis.

### Primary analyses

Biomarkers were log-transformed because of skewed distributions. First, group-level comparisons were performed with analyses of covariance (ANCOVAs), and subsequent post hoc pair-wise comparisons, to compare log-transformed CSF biomarker levels between former players with NBD, without NBD, and unexposed individuals without NBD. Subsequent analyses using NBD factor scores were exclusively conducted within the entire cohort of former American football players (with and without NBD diagnosis) and not within the cohort of unexposed individuals (due to consistently low factor scores in this cohort). We standardized NBD factor scores (*z*-scores) and multivariable linear regression models were used to assess the associations between log-transformed inflammatory biomarkers (IL-1β, IL-6, IL-8, IL-10, CRP, TNF-α) and NBD measures (explosivity, emotional dyscontrol, affective lability, impulsivity, NBD total score). Separate multivariable linear regression models tested the relationships between RHI exposure level (total years of football, CHII linear acceleration) and log-transformed CSF inflammatory biomarker concentrations.

### Sensitivity analyses

Previous studies from the DIAGNOSE CTE Research Project found stronger effects for biomarkers in older participants [21, 22]. Therefore, we subsequently performed analyses described above within age-stratified groups (< 60, ≥ 60 years old). This cut-off point is close to the median age of the cohort (57 years) and has been used in previous studies using the same subject cohort.

Since plasma is a less-invasive biomarker collection method, we explored whether similar effects are found for the same inflammatory biomarkers but in plasma. We examined the correlation between concentrations of each log-transformed plasma and CSF inflammatory biomarker (Pearson’s correlation coefficient). Group-level differences between the three NBD groups were measured with ANCOVAs, and multivariable linear regression models were employed to assess the relationship between plasma inflammatory biomarkers and NBD symptoms within former football players.

To explore another potential pathway related to NBD in addition to neuroinflammation, we examined the effects of plasma neurofilament light (NfL) chain protein as a marker for neuroaxonal damage and neurodegeneration. Due to good correlations between plasma and CSF NfL levels in this sample (Spearman correlation *r* = 0.64, 95% CI 0.53–0.73), and the greater availability of plasma samples, we selected plasma NfL as the marker for analysis. Specific procedure and assay details are provided in Additional file 1. Again, log-transformed plasma NfL concentrations were compared between the three groups, and we calculated the association between plasma NfL and NBD factor scores within the cohort of former football players. These models were further analyzed within age-stratified groups (< 60 and ≥ 60 years old), because NfL is known to have a significant age effect [23].

To investigate whether biomarkers are associated not only with NBD but also with broader clinical TES symptoms in former football players, we also examined biomarker effects on cognition. We employed separate multivariable linear models using relevant log-transformed biomarkers (previously identified significant biomarkers) as predictors, and two cognitive factor scores as outcome measures: Verbal Memory and Executive Functioning and Processing Speed (see detailed information on cognitive factor scores in Additional file 1). We then re-analyzed linear models between relevant log-transformed inflammatory biomarkers, and NBD factor scores, while including a cognitive factor score as a covariate. This allows us to determine the direct effects of biomarkers on NBD factor scores independent of cognitive performance. Models were performed only within the cohorts of interest (entire cohort or age-stratified), based on significance in previous models.

Some participants were receiving antidepressants, which could confound the relationship between inflammatory biomarkers and NBD, so relevant models were repeated with antidepressant use as an extra covariate. In addition, two participants were identified as having a diagnosis of a systemic autoimmune disorder (rheumatoid arthritis *N* = 1, Crohn’s disease *N* = 1), and one participant was receiving systemic corticosteroid treatment. To test whether these individuals affected the discovered relationships between inflammatory biomarkers and NBD, models were repeated with these cases excluded.

### Additional statistical details

To control for false discovery rate (FDR) when conducting multiple comparisons, we used the Benjamini–Hochberg method for each separate research question or Tukey post hoc testing for ANCOVA models. Demographic results are presented as frequency + percentage (%), mean + standard deviation (SD), and median with interquartile range (IQR). Chi-square tests or analyses of variance (ANOVA) measured differences in individual characteristics between the three study groups (Table 1). ANCOVA outcomes are presented with *F*-values, including degrees of freedom. Regression results include standardized beta (*β*), including 95% confidence intervals (CI). Both uncorrected *p*-value (uncorr-*p*), and FDR-corrected *p*-value (FDR-p) are displayed. For ANCOVAs, we included covariates: age, BMI, antidepressant use and rFSRP score to correct for potential confounds. For regression models, we included the covariates: age, self-identified race, BMI, total AUDIT score, *APOE-e4* allele carrier status, rFSRP score, and sleep apnea. Models with cognition as an outcome measure included years of education additionally. Due to missing CSF and/or blood samples and missing NBD and/or cognitive scores, the sample sizes of the models were reduced (Additional file 1: Table A.1). Analyses were done in RStudio (version 4.2.1), using the *lm,* and *aov* functions*.*

**Table 1:** Sample characteristics

	Former football players, NBD + (*n* = 104)	Former football players, NBD− (*n* = 76)	Unexposed individuals, NBD− (*n* = 55)
*Demographics*			
Age, mean (SD) years	58.2 (7.7)	58.2 (8.7)	59.8 (8.3)
Age < 60 years, *n* (%)	68 (65.4)	41 (53.9)	26 (47.3)
Age ≥ 60 years, *n* (%)	36 (34.6)	35 (46.1)	29 (51.8)
Education, mean (SD) years^**^	16.5 (1.0)	17.1 (1.9)	17.5 (3.5)
Race (self-identified), *n* (%)			
Black or African-American	39 (37.5)	24 (31.6)	19 (34.5)
White	63 (60.6)	51 (67.1)	35 (63.6)
Native Hawaiian or other Pacific Islander	0 (0.0)	1 (1.3)	1 (1.8)
Multiple races	2 (1.9)	0 (0.0)	0 (0.0)
Ethnicity, *n* (%)			
Hispanic or Latino	2 (1.9)	1 (1.3)	0 (0.0)
*Football*			
College, *n* (%)	36 (34.6)	24 (31.6)	–
Professional, *n* (%)	68 (65.4)	52 (68.4)	–
Total years of football play, mean (SD) years	16.3 (4.5)	15.2 (4.1)	–
Cumulative head impact index (CHII) – linear acceleration, ^a^ mean (SD) *g*	174,782 (54,303)	167,482 (50,717)	–
*Alcohol use*			
Alcohol use Disorders identification test score, ^b^ mean (SD)^*^	5.5 (6.5)	4.5 (4.3)	2.9 (3.2)
*Cardiovascular risk*			
Body mass index, mean (SD)^*^	33.0 (4.6)	32.0 (4.7)	30.9 (4.7)
Revised Framingham Stroke Risk Profile, ^c^ mean (SD)^**^	0.03 (0.03)	0.03 (0.04)	0.05 (0.04)
*Sleep apnea*			
Self-reported history of sleep apnea, *n* (%)^*^	40 (38.5)	22 (28.9)	10 (18.2)
*APOE genotype*			
*ε4* allele*,* *n* (%) present^*^	38 (36.5)	15 (19.7)	10 (18.2)
*Medication*			
Antidepressants use, *n* (%)^***^	30 (28.8)	6 (7.9)	2 (3.6)
*Neurobehavioral dysregulation (raw factor scores)*^*d*^			
Explosivity^***^	0.49 (0.16)	0.33 (0.07)	0.29 (0.04)
Emotional dyscontrol^***^	0.68 (0.19)	0.43 (0.11)	0.36 (0.05)
Affective lability^***^	0.42 (0.20)	0.28 (0.12)	0.25 (0.10)
Impulsivity^***^	0.56 (0.15)	0.41 (0.08)	0.37 (0.07)
NBD total score^***^	53.6 (13.4)	36.1 (6.8)	31.8 (4.4)

Characteristics of three study groups: (1) former American football players, with NBD diagnosis (NBD +), (2) former American football players without NBD diagnosis (NBD−), (3) healthy individuals without exposure to TBI or RHI, without NBD diagnosis. ^a^CHII linear acceleration is an estimate of the total linear acceleration (measured in g-force) that a football player has been exposed to during his career (range 47,298–337,689). ^b^The Alcohol Use Disorders Identification Test is a 10-item screening tool, scores from 8–14 indicate harmful consumption, > 15 suggests the likelihood of alcohol dependency. ^c^The Revised Framingham Stroke Risk Profile served as a vascular composite risk score, including measures for systolic blood pressure, use of antihypertensive medication, cardiovascular disease, current smoking status, atrial fibrillation, and diabetes. ^d^Range values for NBD factor scores: explosivity (0.27–0.90), emotional dyscontrol (0.33–1.0), affective lability (0.2–1.0), impulsivity (0.29–0.96), NBD total score (27.2–87.2). *uncorr-*p* < 0.05, **uncorr-*p* < 0.01, ***uncorr-*p* < 0.001; *p*-values from group-level ANOVAs or Chi-square tests.

## Results

### Sample characteristics and group comparisons

Table 1 provides the characteristics of the sample of former American football players with NBD (*n* = 104), without NBD (*n* = 76), and unexposed individuals without NBD (*n* = 55). After applying FDR-correction, there were no significant differences in CSF inflammatory biomarkers between the three groups (Table 2), neither after stratification by age (Additional file 1: Tables B.1, B.2)*.* Post hoc testing demonstrated higher CSF IL-6 levels in former football players with NBD compared to players without NBD (1.73 pg/mL, 1.42 pg/mL, *p* = 0.032), but not compared to unexposed individuals (*p* = 0.071) (Additional file 1: Table B.1, B.2).

**Table 2:** Inflammatory biomarkers

	Former football players NBD +	Former football players NBD-	Unexposed individuals NBD-	uncorr-*p*	FDR*-p*
*CSF biomarkers*	*n = 76*	*n = 61*	*n = 41*		
CSF interleukin-1β, mean (SD) pg/mL	0.12 (0.09)	0.10 (0.08)	0.11 (0.09)	*0.358*	*0.716*
CSF interleukin-6, mean (SD) pg/mL	1.73 (0.93)	1.42 (0.57)	1.43 (0.68)	0.020*	*0.120*
CSF interleukin-8, mean (SD) pg/mL	47.1 (13.2)	48.7 (12.9)	47.2 (14.9)	*0.549*	*0.824*
CSF interleukin-10, mean (SD) pg/mL	0.09 (0.08)	0.08 (0.07)	0.08 (0.06)	*0.704*	*0.845*
CSF C-reactive protein, median (IQR) pg/mL	5390 (9778)	4370 (6267)	4730 (6910)	*0.266*	*0.716*
CSF tumor necrosis factor-α, mean (SD) pg/mL	0.13 (0.13)	0.13 (0.12)	0.14 (0.15)	*0.924*	*0.924*
*Plasma biomarkers*	*n = 102*	*n = 75*	*n = 54*		
Plasma interleukin-1β, mean (SD) pg/mL	0.06 (0.19)	0.05 (0.06)	0.07 (0.12)	*0.357*	*0.625*
Plasma interleukin-6, median (IQR) pg/mL	0.72 (1.0)	0.65 (0.0)	0.80 (1.0)	*0.167*	*0.390*
Plasma interleukin-8, median (IQR) pg/mL	4.52 (3.0)	4.66 (2.0)	4.27 (3.0)	*0.605*	*0.706*
Plasma interleukin-10, median (IQR) pg/mL	0.23 (0.0)	0.28 (0.0)	0.25 (0.0)	*0.491*	*0.687*
Plasma C-reactive protein, median (IQR) mg/L	1.76 (0.27)	1.11 (0.19)	1.90 (0.20)	0.044*	*0.154*
Plasma tumor necrosis factor-α, mean (SD) pg/mL	2.28 (1.0)	2.08 (0.78)	2.43 (0.74)	0.022*	*0.154*
Plasma neurofilament light, mean (SD) pg/dL	11.9 (9.5)	10.9 (6.3)	11.4 (6.6)	*0.973*	*0.973*

Mean or median biomarker levels within three groups (not log-transformed), including standard deviation (SD) or interquartile range (IQR). Analysis of covariance (ANCOVA) assessed differences in log-transformed cerebrospinal fluid (CSF) and plasma biomarkers between three groups. Models included covariates: age, BMI, rFSRP, and antidepressant use. Uncorrected and FDR-corrected p-values are presented. Full ANCOVA results can be found in Additional file 1: Tables B.1, B.2. * = *p*-values ≤ 0.05

### CSF inflammatory biomarkers and NBD factor scores

**Fig. 1:**
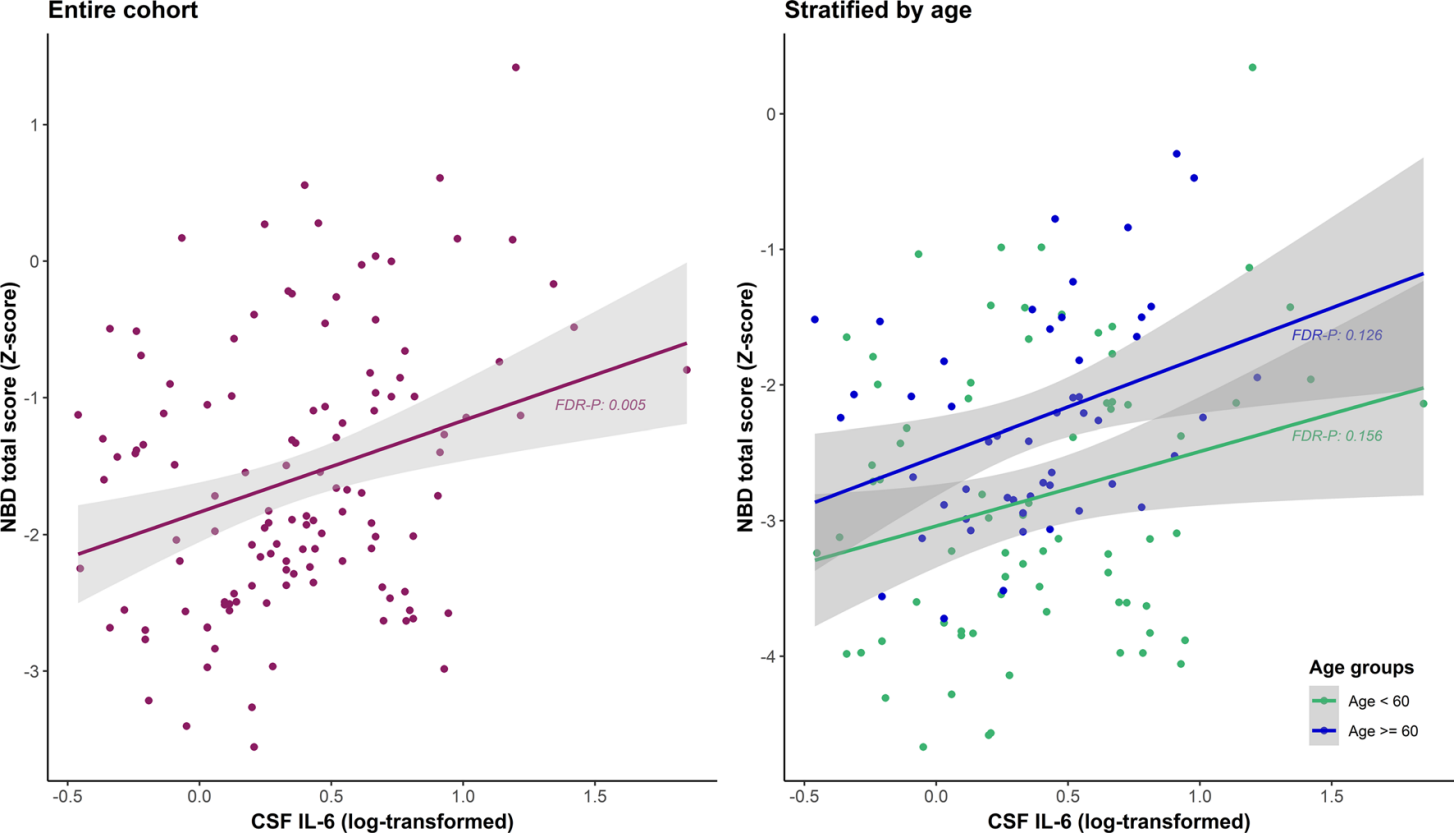
Adjusted associations between CSF IL-6 and NBD total score. Partial regression plots with results from the entire cohort and age-stratified cohorts of former American football players, adjusted for covariates: age, BMI, race, total AUDIT score, *APOE-ε4* carrier, revised Framingham Stroke Risk Profile score, and sleep apnea. Regression results were: *β*: 0.286, 95%: CI 0.120–0.452, FDR-p: 0.005 (entire cohort), *β*: 0.247, 95% CI: 0.013–0.481, FDR-p: 0.156 (age < 60), *β*: 0.366, 95% CI: 0.089–0.642, FDR-p: 0.126 (age ≥ 60)

Within the cohort of former football players, significant associations were found between CSF IL-6 and emotional dyscontrol, affective lability, impulsivity, and the NBD total score (Table 3, Fig. 1). The effect sizes of these relationships were relatively similar in both age groups; only impulsivity showed a stronger relationship in the older age group. Furthermore, a significant association was found between CSF CRP and impulsivity (*β* = 0.431, 95% CI 0.161–0.701, FDR-*p* = 0.017) only within the ≥ 60 years age group. There were no significant associations between any of the other inflammatory biomarkers (CSF IL-1β, IL-8, IL-10, TNF-α) and NBD factor scores (Additional file 1: Tables C.1–C.5).

**Table 3:** Associations between CSF IL-6 and NBD factor scores

CSF IL-6 (log)	β (95% CI)	uncorr-p	FDR-p
*Entire cohort*			
Explosivity	0.170 (− 0.001–0.341)	0.051	0.294
Emotional dyscontrol	0.240 (0.068–0.413)	0.007**	0.041*
Affective lability	0.276 (0.107–0.446)	0.002**	0.012*
Impulsivity	0.294 (0.128–0.460)	0.0006***	0.004**
NBD total	0.286 (0.120–0.452)	0.0009***	0.005**
*Age < 60*			
Explosivity	0.130 (− 0.115–0.376)	0.293	0.455
Emotional dyscontrol	0.184 (− 0.058–0.425)	0.133	0.336
Affective lability	0.286 (0.063–0.509)	0.013*	0.156
Impulsivity	0.245 (0.007–0.482)	0.043*	0.153
NBD total	0.247 (0.013–0.481)	0.039*	0.156
*Age ≥ 60*			
Explosivity	0.247 (− 0.033–0.527)	0.083	0.455
Emotional dyscontrol	0.357 (0.066–0.648)	0.018*	0.106
Affective lability	0.191 (− 0.122–0.504)	0.225	0.959
Impulsivity	0.421 (0.153–0.690)	0.003**	0.017*
NBD total	0.366 (0.089–0.642)	0.011*	0.126

Results from multivariate linear regression models between cerebrospinal (CSF) concentrations of Interleukin (IL)-6 (log-transformed) and neurobehavioral dysregulation (NBD) factor scores, within the entire cohort and age-stratified cohorts of former American football players. Models included covariates: age, BMI, race, total AUDIT score, *APOE-ε4* carrier, revised Framingham Stroke Risk Profile score, and sleep apnea. Results are presented as standardized beta (*β*), with uncorrected and FDR-corrected *p*-values. * = *p*-values ≤ 0.05, ** = *p*-values ≤ 0.01, *** = *p*-value ≤ 0.001

### RHI exposure and CSF inflammatory biomarkers

As demonstrated in Additional file 1: Tables D.1, D.2, total years of football and CHII were not significantly related to concentrations of inflammatory biomarkers, after applying the FDR correction for multiple comparisons.

### Sensitivity analyses: plasma inflammatory biomarkers

Only a strong significant correlation was found between CSF and plasma CRP (*r* = 0.729, 95% CI 0.652–0.719, *p* ≤ 0.001). The correlation between CSF and plasma IL-8 was weak (*r* = 0.233, 95% CI 0.089–0.368, *p* = 0.002). No correlations were found between plasma/CSF IL-1β, IL-6, IL-10, and TNF-α (Additional file 1: Table E.1). Plasma inflammatory biomarkers did not differ between the three groups (Table 3), only post hoc testing revealed lower plasma TNF-α in former players without NBD compared to unexposed participants (*p* = 0.017). Plasma inflammatory biomarkers were not associated with NBD factor scores (Additional file 1: Tables F.1).

### Sensitivity analyses: plasma NfL and neurobehavioral dysregulation

**Fig. 2:**
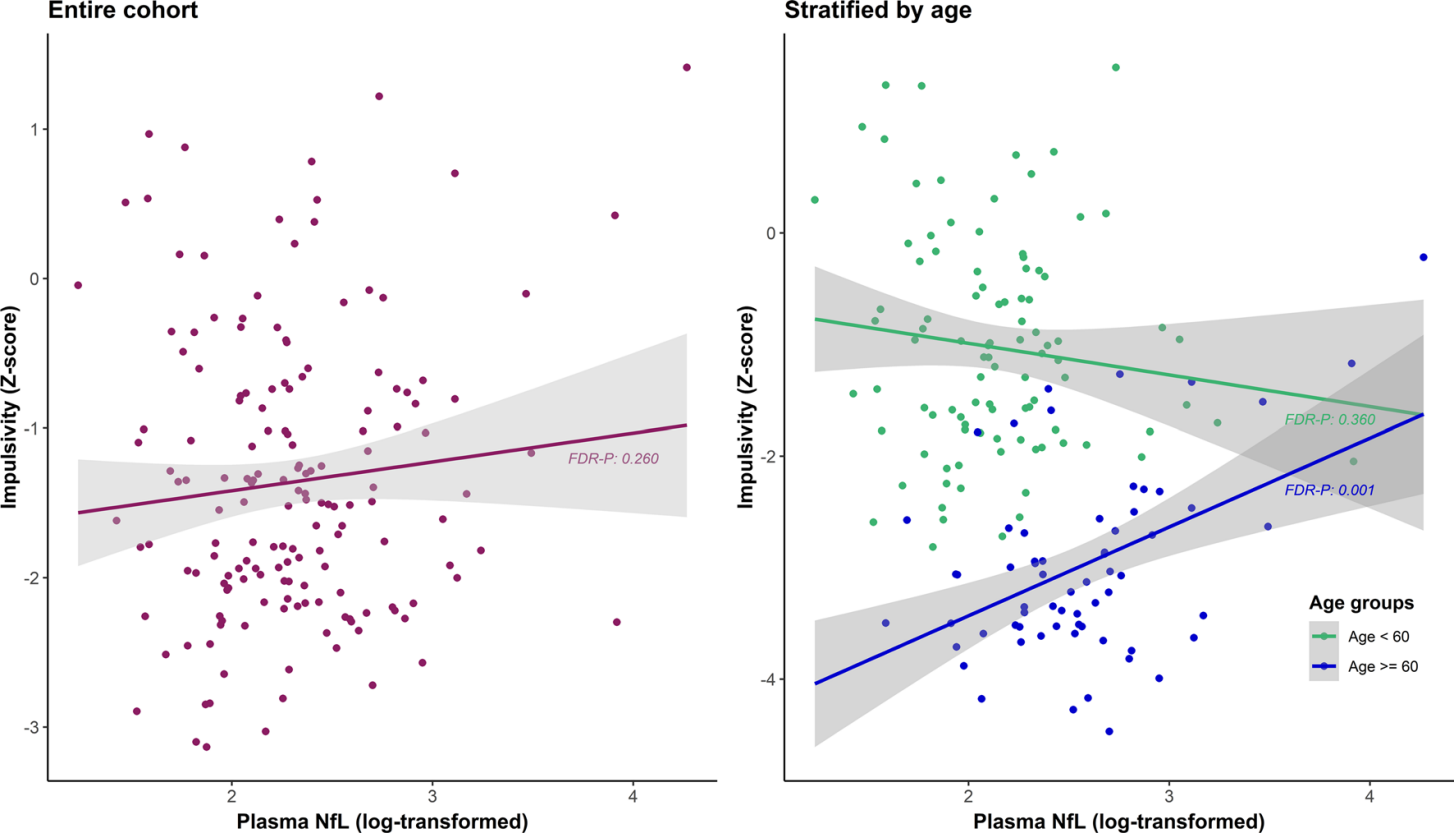
Adjusted associations between Plasma NfL and Impulsivity. Partial regression plots with results from the entire cohort and age-stratified cohorts of former American football players, adjusted for covariates: age, BMI, race, total AUDIT score, *APOE-ε4* carrier, revised Framingham Stroke Risk Profile score, and sleep apnea. Regression results were: *β*: 0.126, 95% CI: − 0.048–0.300, FDR-p: 0.260 (entire cohort), *β*: − 0.138, 95% CI: − 0.359–0.082, FDR-p: 0.360 (age < 60), *β*: 0.488, 95% CI: 0.254–0.723, FDR-p: 0.001 (age ≥ 60)

Only in the older age group, significant relationships were found between plasma NfL and emotional dyscontrol, plasma NfL and impulsivity, and plasma NfL and NBD total score (Table 4, Fig. 2). This relationship was not found in the entire cohort or the < 60 years cohort.

**Table 4:** Associations between plasma NfL and NBD factor scores

Plasma NfL (log)	*β* (95% CI)	uncorr-*p*	FDR-*p*
*Entire cohort*			
Explosivity	0.089 (− 0.084–0.263)	0.311	0.311
Emotional dyscontrol	0.207 (0.031–0.382)	0.021*	0.105
Affective lability	0.093 (− 0.086–0.273)	0.306	0.311
Impulsivity	0.126 (− 0.048–0.300)	0.156	0.260
NBD total	0.160 (− 0.013–0.333)	0.070	0.174
*Age < 60*			
Explosivity	0.019 (− 0.205–0.243)	0.868	0.868
Emotional dyscontrol	0.097 (− 0.126–0.320)	0.390	0.557
Affective lability	0.222 (0.006–0.438)	0.044*	0.110
Impulsivity	− 0.138 (− 0.359–0.082)	0.216	0.360
NBD total	0.077 (− 0.145–0.298)	0.494	0.618
*Age ≥ 60*			
Explosivity	0.206 (− 0.050–0.461)	0.112	0.224
Emotional dyscontrol	0.402 (0.143–0.661)	0.003**	0.015*
Affective lability	− 0.078 (− 0.352–0.196)	0.570	0.633
Impulsivity	0.488 (0.254–0.723)	0.0001***	0.001**
NBD total	0.333 (0.079–0.586)	0.011*	0.037*

Sensitivity analyses: results from multivariate linear regression models between plasma concentrations of neurofilament light chain (NfL) (log-transformed) and neurobehavioral dysregulation (NBD) factor scores, within the entire cohort and age-stratified cohorts of former American football players. Models included covariates: age, BMI, race, total AUDIT score, *APOE-ε4* carrier, revised Framingham Stroke Risk Profile score, and sleep apnea. Results are presented as standardized beta (*β*), with uncorrected and FDR-corrected *p*-values. * = *p*-values ≤ 0.05, ** = *p*-values ≤ 0.01, *** = *p*-value ≤ 0.001

### Sensitivity analysis: systemic inflammatory diseases and medication use

Results remained unaffected after excluding three individuals with diseases or medications that potentially affect the systemic immune system. After including antidepressant use as a covariate in the models, the relationship between CSF IL-6 and affective lability, impulsivity, and NBD total score remained significant. The relationship between IL-6 and emotional dyscontrol, as well as IL-6, CRP, and impulsivity in the older age category lost significance (Additional file 1: Tables H.1, H.2).

### Sensitivity analysis: biomarkers, NBD, and cognition

No associations were found between CSF IL-6 or CRP and cognitive factor scores (Verbal Memory and Executive Functioning and Processing Speed) (Table 5). When re-examining the relationship between inflammatory markers and NBD factor scores, while including cognitive factor scores as covariates, we found that the association between CSF IL-6 and emotional dyscontrol was attenuated (FDR-p = 0.084 and 0.066 for Verbal Memory and Executive Functioning/Processing Speed, respectively). Significant relationships remained between CSF IL-6 and affective lability, impulsivity, and NBD total score. In the ≥ 60 years cohort, the relationship between CSF CRP and impulsivity also remained significant (Additional file 1: Tables G.1, G.2).

**Table 5:** Associations between biomarkers of interest and cognition

	*β* (95% CI)	uncorr-*p*	FDR-*p*
CSF IL-6			
*Entire cohort*			
Verbal memory	− 0.101 (− 0.290 to 0.087)	0.288	0.576
Executive + speed	− 0.069 (− 0.236 to 0.097)	0.410	0.547
*Age ≥ 60*			
Verbal memory	− 0.045 (− 0.402 to 0.311)	0.798	0.798
Executive + speed	− 0.164 (− 0.432 to 0.105)	0.225	0.450
CSF CRP			
*Age ≥ 60*			
Verbal memory	0.187 (− 0.144 to 0.518)	0.260	0.576
Executive + speed	0.027 (− 0.229 to 0.284)	0.830	0.830
Plasma NfL			
*Age ≥ 60*			
Verbal memory	0.082 (− 0.212 to 0.375)	0.581	0.775
Executive + speed	− 0.313 (− 0.540 to − 0.086)	0.008**	0.032*

Sensitivity analyses: results from multivariate linear regression models between concentrations of log-transformed biomarker concentration of interest and cognitive factor scores; Verbal Memory and Executive Functioning and Processing Speed. Models were performed within cohorts of interest, based on previous significance with NBD factor scores. Models included covariates: age, BMI, race, total AUDIT score, *APOE-ε4* carrier, revised Framingham Stroke Risk Profile score, sleep apnea, and years of education. Results are presented as standardized beta (*β*), with uncorrected and FDR-corrected *p*-values

* = *p*-values ≤ 0.05, ** = *p*-values ≤ 0.01

**Fig. 3:**
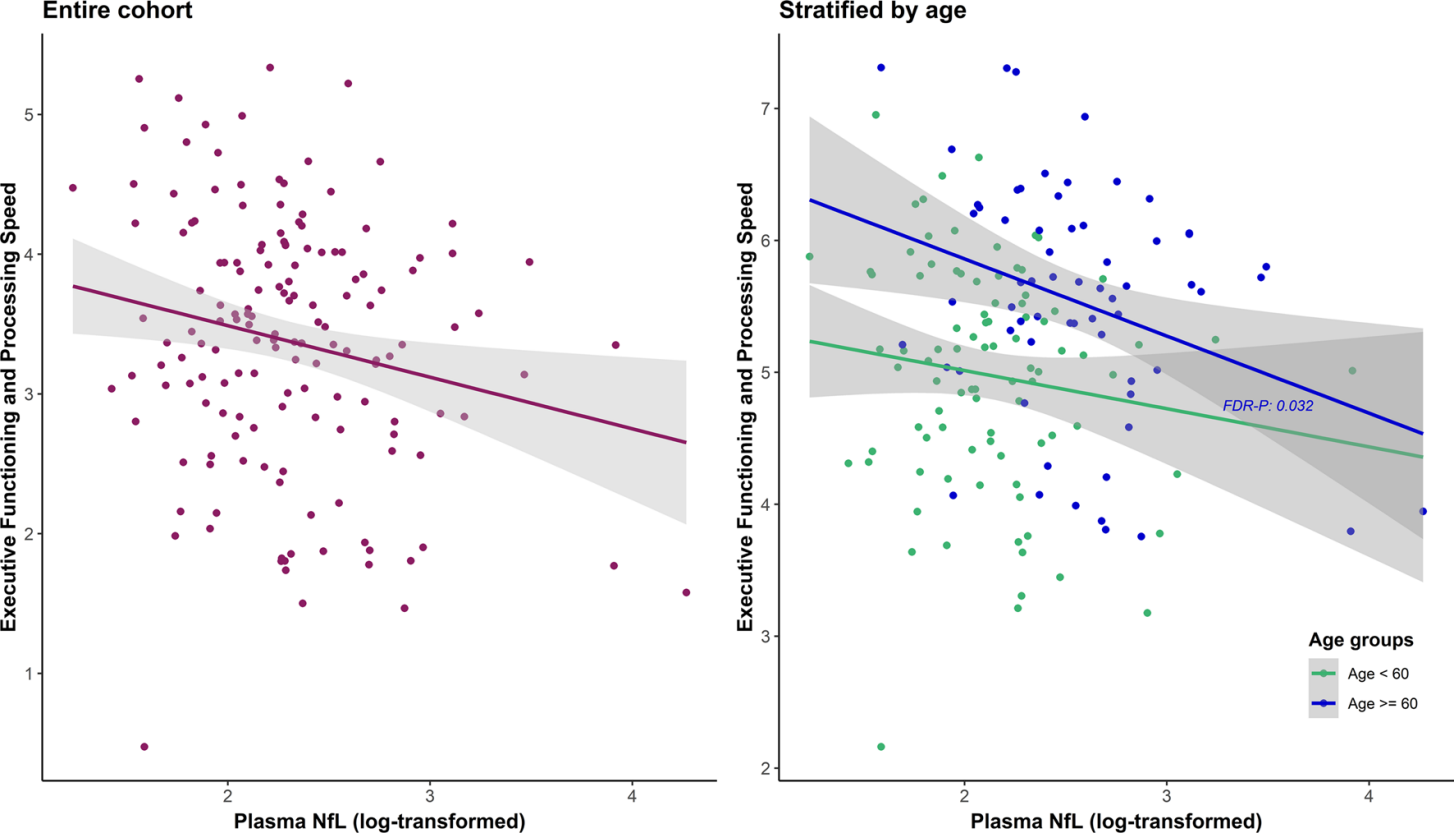
Adjusted associations between Plasma NfL and Executive Functioning and Processing Speed. Partial regression plots with results from the entire cohort and age-stratified cohorts of former American football players, adjusted for covariates: age, BMI, race, total AUDIT score, *APOE-ε4* carrier, revised Framingham Stroke Risk Profile score, sleep apnea, and years of education. Regression results within the age ≥ 60 cohort: *β*: − 0.313, 95% CI: − 0.540– − 0.086, FDR-p: 0.03

As displayed in Table 5 and Fig. 3, a significant negative relationship was found between plasma NfL and Executive Functioning and Processing Speed, but not between plasma NfL and Verbal Memory. The relationships between plasma NfL and emotional dyscontrol, impulsivity, and NBD total score remained significant after adding Verbal Memory as a covariate. When adding Executive Functioning and Processing Speed as covariate, the relationship between plasma NfL and emotional dyscontrol and total NBD score lost significance (FDR-P = 0.100 and 0.110, respectively), but remained significant between plasma NfL and impulsivity (*β* = 0.484, 95% CI 0.225–0.743, FDR-p = 0.004) (Additional file 1: Tables G.1, G.2).

## Discussion

In this study, we investigated neuroinflammatory markers in former professional and college American football players and the relationship with the neuropsychiatric syndrome, neurobehavioral dysregulation (NBD). Overall, former football players diagnosed with NBD did not have significantly different levels of CSF or plasma inflammatory biomarkers concentrations compared to asymptomatic unexposed individuals. However, CSF IL-6 levels differed between former football players with and without NBD diagnosis and we observed significant associations between levels of CSF IL-6 and elevated scores on specific NBD measures (emotional dyscontrol, affective lability, impulsivity, and overall neurobehavioral dysregulation). This association was not observed for explosivity, and a sensitivity analysis found that the relationship with emotional dyscontrol was dependent of cognitive performance or the use of antidepressants. CSF IL-6 was specifically associated with affective lability, impulsivity, and overall NBD and did not correlate with cognitive performance. Furthermore, in former football players ≥ 60 years, higher concentrations of plasma NfL were associated with more self-reported symptoms of emotional dyscontrol, impulsivity, and overall NBD. Elevated NfL levels were also associated with poorer executive functioning and processing speed. These results collectively show that NBD symptoms among former American football players may be associated with an altered neuroinflammatory response and that, in later life, specific NBD symptoms may be part of a broader clinical syndrome that potentially involves both neuroinflammation and neurodegeneration. The absence of an association between plasma IL-6 and NBD indicates that the inflammatory link is specifically central in nature, rather than systemic.

Our findings suggest that, while multiple biomarkers for neuroinflammation did not show a consistent relationship with NBD, IL-6 demonstrated a more robust and specific association. IL-6 is a pro-inflammatory cytokine that plays a pivotal role in inflammatory responses within the central nervous system. It exhibits pleiotropic features and is produced by various cell types including microglia, astrocytes, and neurons [24, 25]. The role of IL-6 is multifaceted, involving the regulation of acute-phase inflammatory responses as well as participation in adaptive immune responses through multiple pathways, potentially contributing to chronic ongoing inflammation [24, 25]. Overall, IL-6 appears to be a more general and sensitive marker capable of detecting subtle changes and correlations [25]. It should be noted that the studied population is considered relatively "healthy" and not expected to show severe systemic inflammatory responses as seen in primary inflammatory diseases. Consequently, the finding that only IL-6 is associated with NBD symptoms in this population may not be surprising, as this requires a sensitive marker.

The association between IL-6 and neuropsychiatric symptoms has been assessed in a variety of disorders. In the context of AD, studies have demonstrated that increased serum concentrations of IL-6 are associated with overall neuropsychiatric symptoms or higher odds of apathy [13, 26, 27]. Conversely, other studies did not find significant relationships between CSF or serum IL-6 levels and neuropsychiatric symptoms in AD patients [27, 28] and a recent study reported that even lower CSF levels of IL-6 were associated with symptoms of depression and anxiety [29]. In frontotemporal dementia (FTD), patients with disinhibition had higher levels of plasma IL-6 compared to patients without disinhibition [30]. Elevations in IL-6 have also been linked to various primary psychiatric syndromes and corresponding symptoms. Two meta-analyses have demonstrated that CSF concentrations of IL-6 are elevated in patients with major depressive disorder compared to controls [31, 32]. In contrast, a recent study that focused on a more homogeneous sample of patients with recent depression did not observe significant elevations in CSF IL-6 levels nor associations with the severity of depressive symptoms [33]. Elevated levels of IL-6 in blood have been found in patients with bipolar disorder, most strongly during the acute mania phase [34]. Additionally, higher levels of serum IL-6 have been found in individuals with intermittent explosive disorder and were positively correlated with the level of aggressive symptoms [35]. Overall, despite conflicting findings, there appears to be an association between elevated levels of central and peripheral IL-6 and the presence of diverse neuropsychiatric symptoms in a variety of neurological and psychiatric conditions. This suggests that elevated IL-6 levels may reflect an altered emotional state commonly observed in these disorders. Consequently, IL-6 has the potential to serve as a sensitive but non-specific marker.

NBD is proposed to be a distinct syndrome encompassing neuropsychiatric symptoms reported in athletes exposed to RHI. The specific components comprising NBD may share overlapping features with symptoms observed in other neurological and psychiatric disorders, although there seem to be notable differences. For instance, symptoms similar to NBD can be observed in individuals who have experienced a single TBI, but these symptoms usually remain stable [36, 37]. Impulsive behavior is also recognized as part of behavioral disinhibition in the behavioral variant of FTD [38], while other behavioral changes such as stereotypic behavior or hyperorality are not noted in NBD. Furthermore, NBD exhibits overlap with mild behavioral impairment, which refers to the neuropsychiatric symptoms in early AD [39, 40], but these symptoms seem to develop later in life than NBD in individuals exposed to substantial RHI. Overlap also exists between NBD and corresponding symptoms of primary psychiatric diseases. According to the fifth edition of the Diagnostic and Statistical Manual of Mental Disorders (DSM-V), explosivity and impulsive behavior are key features of intermittent explosive disorder, while tearfulness, irritability, and agitation can be present in major depressive disorder or bipolar disorder [41]. Distinguishing neuropsychiatric syndromes can be difficult due to symptom overlap. This difficulty applies to both primary psychiatric syndromes and differentiating NBD in individuals exposed to RHI. Complexity increases when coexisting or pre-existing psychiatric syndromes are present. Furthermore, rating scales used to assess symptom severity in these syndromes often contain items that share similarities with NBD symptoms. Consequently, we decided not to include psychiatric disease diagnoses or scores from rating scales measuring other neuropsychiatric symptoms, such as depression, hopelessness, apathy, or anxiety in the analyses of our study. Further investigations are necessary to unravel the construct of NBD and its association with other neuropsychiatric syndromes or symptoms.

It remains unclear what the etiology is of the neuroinflammatory association observed in this study in relation to NBD and whether this is directly linked to exposure to RHI. Notably, CSF IL-6 concentrations were not significantly higher in symptomatic former football players compared to asymptomatic unexposed individuals. It may be possible that this is related to limited power due to the small sample size of unexposed individuals with CSF available. This is supported by the finding (see Table 2) that the former players without NBD had almost identical CSF IL-6 means and SDs as the unexposed controls, although there was only a significant difference between the former players with NBD and those without NBD, but not between the former players with NBD and the controls.

Alterations in IL-6 have previously been observed in studies investigating TBI or RHI. Elevated levels of IL-6 were observed following mild TBI and were associated with poorer outcomes [42–45]. Military personnel and veterans who sustained ≥ 3 TBI had higher serum IL-6 concentrations compared to controls, and these elevated IL-6 levels were associated with symptoms of posttraumatic stress disorder (PTSD) [46]. One recent study found that male individuals with previous RHI exposure through contact or collision sport, of whom the majority met the criteria for TES, had higher levels of plasma IL-6 compared to both healthy controls and AD patients [47]. Further evidence for chronic microglial activation in RHI-exposed individuals comes from neuropathology studies as well as translocator protein (TSPO)-positron emission tomography (PET) studies [5, 7, 48, 49]. Neuroinflammation seems to play a substantial role in the complex pathways following RHI. Several experimental mice studies testing anti-inflammatory treatments after RHI demonstrated reduced inflammatory responses and improvement in clinical performance [50–52]. This neuroinflammatory response can be short-term but can also lead to persistent chronic activation, with associated astrogliosis and p-tau pathology [6, 53], or behavioral changes [8, 54]. Another study showed evidence of neuroinflammation associated with microvascular injury and p-tau pathology [4]. The directionality of the relationship between neuroinflammation and p-tau is debatable, because p-tau may also induce neuroinflammation and progressive cell dysfunction. This highlights the complex relationship between neuroinflammation and neurodegenerative diseases, raising questions about whether neuroinflammation after RHI is a causative factor or a consequence of p-tau pathology and neurodegeneration [55, 56].

Neurodegeneration, reflected by plasma NfL concentrations, also appears to be linked to NBD in former football players. Plasma NfL serves as a biomarker for neuroaxonal damage and is elevated in multiple central nervous system diseases, including several neurodegenerative disorders [23]. In these disorders, NfL levels demonstrate good discriminative ability in both plasma and CSF, along with correlations to clinical performance [57–59]. The association between plasma NfL and NBD scores solely within the older age group is not unexpected. Plasma NfL levels increase with advancing age, as does the risk of developing neurodegenerative processes [23, 60], thus implying a higher likelihood of detecting an effect within this older age category. Furthermore, the relationship between plasma NfL levels and NBD scores showed a significant relationship with executive function and processing speed, suggesting the existence of a clinical syndrome characterized by executive and behavioral dysregulation. This syndrome may be related to chronic axonal degeneration later in life. However, further investigation using longitudinal biomarkers and clinical data is warranted to comprehensively examine these associations and clinical trajectories.

We found no significant associations between CSF IL-6 and cognitive performance and found that the relationship between IL-6 and most NBD factor scores was not influenced by cognitive performance. Previous studies have yielded mixed results regarding the association between IL-6 and cognition. Studies observed altered peripheral IL-6 levels in patients with mild cognitive impairment and AD dementia, but this evidence was less strong for CSF IL-6 [61]. Other studies found no links between high IL-6 and worse cognitive performance [62, 63]. Our findings suggest that IL-6 specifically reflects NBD, unlike NfL, which appears to reflect a more general clinical syndrome. Nevertheless, since our data are cross-sectional, these hypotheses should be interpreted with caution and require further investigation in longitudinal studies.

The lack of association between inflammatory markers and explosivity symptoms in this sample suggests that explosivity may be attributed to other factors. Individuals who have already notable levels of aggression and hostility may tend to participate in youth and/or high school football and are more likely to reach college or professional levels later in life. Further research is needed to discover how these symptoms further exacerbate after a football career and what the role is of other factors, such as poor health behaviors and social determinants of health.

Strengths of this study include a substantial sample size of football players and CSF and plasma samples, along with comprehensive clinical and neuropsychiatric data. However, it is important to consider the limitations of this study when interpreting the findings. The study population consisted of male former American football players. As a result, the generalizability of these findings may be limited. Since we did not include symptomatic reference groups, future studies should also focus on non-RHI exposed individuals with symptoms related to NBD, such as those seen in AD patients with neuropsychiatric symptoms, behavioral variant FTD patients, individuals with neuropsychiatric symptoms following single moderate/severe TBI, or patients with primary psychiatric disease. We also did not present results from a medication-free population and did not account for the use of medication other than antidepressants and corticosteroids. For example, we did not account for nonsteroidal anti-inflammatory drug (NSAID) use. Although NSAIDs have been shown to pass the blood–brain barrier, resulting in diluted but detectable levels of NSAIDS in CSF after oral NSAID intake, cytokine levels in CSF did not show significant alterations and we, therefore, expect that NSAID use would not affect our observed CSF results [64]. This study consisted of cross-sectional data; the neuropsychiatric assessments and CSF included only single measurements while these might be prone to dynamic changes.

## Conclusions

We found associations between elevated levels of CSF IL-6 and plasma NfL with certain symptoms of neurobehavioral dysregulation in former American football players. This study adds evidence to the hypothesis that neuropsychiatric changes in former American football players may be multifactorial and associated with various pathologies, and highlights the complex interplay between RHI-exposure, neuroinflammation, neurodegeneration, and neurobehavioral dysregulation. The role and directionality of these pathophysiological processes in combination with other potential biological and non-biological pathways such as white matter degeneration, p-tau pathology, functional network disruptions, health behaviors, and discrepancies in social determinants of health, require evaluation in future studies, using more complex multimodal and longitudinal models.

### Supplementary Information

**Additional file 1: Table A.1.** Sample sizes. **Tables B.1, B.2.** ANCOVA results. **Tables C.1–C.5.** Regression results CSF biomarkers and NBD factor scores. **Tables D.1, D.2.** Regression results RHI exposure and CSF biomarkers. **Tables E.1.** Correlations between CSF and plasma inflammatory markers. **Tables F.1.** Sensitivity analyses (plasma inflammatory biomarkers. **Tables G.1, G.2.** Sensitivity analyses (adjusted for cognition). **Tables H.1, H.2.** Sensitivity analyses (exclusion of cases).

## Data Availability

Data used in this study will be available through the Federal Interagency Traumatic Brain Injury Research (FITBIR) Informatics System, through the National Institutes of Health Center for Information Technology: https://fitbir.nih.gov/content/access-data. Data are also available for qualified investigators through a project-specific data-sharing portal. Interested investigators should contact Dr. Robert A. Stern, bobstern@bu.edu.

## Abbreviations

AD: Alzheimer’s disease
ANCOVA: Analysis of covariance
ANOVA: Analysis of variance
*APOE*: Apolipoprotein E
AUDIT: Alcohol Use Disorders Identification Test
BMI: Body mass index
CHII: Cumulative head impact index
CI: Confidence interval
CRP: C-reactive protein
CSF: Cerebrospinal fluid
CTE: Chronic traumatic encephalopathy
DIAGNOSE CTE: Diagnostics, Imaging, and Genetics Network for the Objective Study and Evaluation of Chronic Traumatic Encephalopathy
dL: Deciliter
DSM-V: Fifth edition of the Diagnostic and Statistical Manual of Mental Disorders
FDR: False discovery rate
FDR-p: False discovery rate corrected p-value
IL: Interleukin
IQR: Interquartile range
L: Liter
mL: Milliliter
NBD: Neurobehavioral dysregulation
NfL: Neurofilament light chain protein
NINDS: National Institute of Neurological Disorders and Stroke
NSAIDs: Nonsteroidal anti-inflammatory drugs
PET: Positron emission tomography
pg: Picogram
p-tau: Phosphorylated tau
rFSRP: Framingham Stroke Risk Profile
RHI: Repetitive head impacts
SD: Standard deviation
TBI: Traumatic brain injury
TES: Traumatic encephalopathy syndrome
TNF-α: Tumor necrosis factor-alpha
TSPO: Translocator protein
uncorr-*p*: Uncorrected *p*-value
VA: Veterans Affairs
*β*: Standardized beta
