# Supplementary material for: Inflammatory biomarkers for neurobehavioral dysregulation in former American football players: findings from the DIAGNOSE CTE Research Project
Source: J Neuroinflammation. 2024 Feb 9;21:46. doi: 10.1186/s12974-024-03034-6 (PMC10854026; doi:10.1186/s12974-024-03034-6)
Supplement: Supplementary file 1 — Additional file 1: Table A.1. Sample sizes. Tables B.1, B.2. ANCOVA results. Tables C.1–C.5. Regression results CSF biomarkers and NBD factor scores. Tables D.1, D.2. Regression results RHI exposure and CSF biomarkers. Tables E.1. Correlations between CSF and plasma inflammatory markers. Tables F.1. Sensitivity analyses (plasma inflammatory biomarkers. Tables G.1, G.2. Sensitivity analyses (adjusted for cognition). Tables H.1, H.2. Sensitivity analyses (exclusion of cases). [file 12974_2024_3034_MOESM1_ESM.docx]

# **Supplementary materials**

# Inflammatory Biomarkers for Neurobehavioral Dysregulation in Former American Football Players – Findings from the DIAGNOSE CTE Research Project

**Suzan van Amerongen**, Surya V. Pulukuri, Fatima Tuz-Zahra, Yorghos Tripodis, Jonathan D. Cherry, Charles Bernick, Yonas E. Geda, Jennifer V. Wethe, Douglas I. Katz, Michael L. Alosco, Charles H. Adler, Laura J. Balcer, Nicholas J. Ashton, Kaj Blennow, Henrik Zetterberg, Daniel H. Daneshvar, Jeffrey J. Iliff, Gail Li, Elaine R. Peskind, Martha E. Shenton**, Eric M. Reiman**, Jeffrey L. Cummings**, Robert A. Stern**, for the DIAGNOSE CTE Research Project Investigators

**denotes shared senior authors

## Contents

Development of the NBD factor scores ....………………………………………………………………………………………….2

Fluid biomarker procedures and assay details ……………………………………………………………………………………3

Development of the Cognitive factor scores….……………………………………………………………………………………4

Table A.1 | Sample sizes….………………………………………………………………………………………………………………….5

Tables B.1 – B.2 | ANCOVA results ………………………………………………………..…………………………………………..7

Tables C.1 – C.5 | Regression results CSF biomarkers and NBD factor scores …………………………………….8

Tables D.1 – D.2 | Regression results RHI exposure and CSF biomarkers ….………………………………………11

Tables E.1 | Correlations between CSF and plasma inflammatory markers ….………………………………….13

Tables F.1 | Sensitivity analyses (plasma inflammatory biomarkers …………………………………………….…..14

Tables G.1 – G.2 | Sensitivity analyses (adjusted for cognition) ……………………………………………………….15

Tables H.1 – H.2 | Sensitivity analyses (exclusion of cases) ………………………………………………………………16

References……………………………………………………………………………………………………………………………………….17

## Development of the NBD factor scores

The baseline assessment for the DIAGNOSE CTE Research Project included multiple questionnaires and/or rating scales to evaluate (neuro)-psychiatric symptoms related to NBD: the Barratt Impulsiveness Scale (BIS-11) (1), the Behavior Rating Inventory of Executive Function-Adult (BRIEF-A) (2), the Brown-Goodwin Lifetime History of Aggression (BGLHA) (3), the Buss-Durkee Hostility Inventory (BDHI) (4), the Center for Neurologic Study-Lability Scale (CNS-LS) (5), the Neuropsychiatric Inventory–Questionnaire (NPI-Q) (6), and the State-Trait Anger Expression Inventory 2 (STAXI-2) (7). The separate items of these scales (N=257) were independently categorized by six different experts into an NBD descriptor listed in the NINDS Consensus Diagnostic Criteria for TES (explosiveness, impulsivity, rage, violent outbursts, having a short fuse, emotional lability) (8), or labeled as ‘none’. Items were removed if the majority of experts voted ‘none’ or there was insufficient item consensus. Certain items were removed because of statistical reasons and informant report items were omitted for improved interpretability. This resulted in 34 expert-selected items from self-report questionnaires/rating scales, that were subjected to confirmatory factor analyses. Psychometric evaluation demonstrated the best fit for a four-factor second-order model with excellent internal consistency for all factors (α ≥ 0.86). Based on expert considerations and item origin, these factors were named: explosivity (10 items from the BRIEF-A and STAXI-2), impulsivity (11 items from the BIS-11 and BRIEF-A), emotional dyscontrol (4 items from the BRIEF-A, reflecting difficulties with regulating emotions), affective lability (3 items from the CNS-LS, reflecting labile tearfulness), with an overlapping “Total NBD Score” (28 items). Factor scores were calculated with equal weighting for items. Due to missing data from two participants, factor scores were available for 178 former football players. A comprehensive explanation and validity of the NBD factor scores can be found elsewhere. (9)

## Fluid biomarker procedures and assay details

## *CSF*

CSF was collected via a lumbar puncture by a qualified professional. The participant was placed in lateral decubitus or sitting position and a 22g or 24g Sprotte atraumatic spinal needle was inserted in the L3-4 or L4-5 interspace. Twenty mL of CSF was collected, of which the majority was aliquoted (portions of 0.5 mL) immediately at the bedside into 1.5 mL polypropylene cryotubes, put on dry ice and stored at -70 degrees. Samples were shipped on dry ice from the study site to the neurochemistry lab at VA Puget Sound, Seattle. CSF was stored at -70° until the analyses. IL-1β, IL-6, IL-8, IL-10, and TNF-α were measured with the V-PLEX Proinflammatory Panel 1 (MSD cat # K15049D) and CRP with V-PLEX Vascular Injury Panel 2 (MSD cat # K15198D). Values that fell below the level of detection or level of quantification were substituted with imputed values randomly assigned within the range of zero and the lower detection limit for each analyte.

## *Plasma*

As part of the DIAGNOSE CTE Research Project, blood was collected through an IV access and centrifuged at 1180g for 15 minutes at 4°C, aliquoted, and stored at -70°C within 90 to 120 minutes of collection. Samples were shipped to the neurochemistry lab of the University of Gothenburg, where plasma assays for NfL were performed using the Neurology 4-Plex on a Single molecule array (Simoa) HD-X analyzer according to instructions from the manufacturer (Quanterix, Billerica, MA). The measurements were performed by board-certified laboratory technicians who were blinded to clinical data. Intra-assay coefficients of variation were below 5%. Plasma inflammatory biomarkers were measured at the neurochemistry lab of VA Puget Sound, Seattle, using panels similar to those described above. Plasma biomarkers were available for 177 former football players (102 with NBD, 75 without NBD) and 54 unexposed individuals.

## Development of the cognitive factor scores

Composite factor scores for cognition were derived through a principal component analysis (PCA) of standardized neuropsychological test scores. Comprehensive details are described in a different paper (10)*.* The PCA was performed among all participants (football players and controls) and resulted in a superior five-factor solution. These five factors were named: (1) Verbal Learning and Memory; (2) Executive Function and Psychomotor Speed; (3) Visual Learning and Memory; (4) Number Span Forward and Backward; and (5) Verbal Fluency. We used factor scores for Verbal Learning and Memory; including scores from the Neuropsychological Assessment Battery (NAB) list learning test (11); immediate, short, and long delay recall, and for Executive Functioning and Processing Speed; including scores from Trail Making Test part A, and B (12), NAB Mazes Test, and the Symbol Digit Modalities Test (13). These cognitive domains are suggested to be mostly affected in CTE. (8) Higher factor scores represent better cognitive performance.

## Table A.1

Sample sizes for the multivariable linear regression models. Sample sizes were reduced after listwise deletion of cases with missing variables.

|  | **Sample Sizes** | | |
| --- | --- | --- | --- |
| **Models** | **Entire cohort (n)** | **Age <60**  **(n)** | **Age ≥60**  **(n)** |
| CSF inflammatory biomarkers ~ NBD factor scores | 124 | 73 | 51 |
| Plasma biomarkers ~ NBD factor scores | 161 | 97 | 64 |
| CSF Inflammatory biomarkers ~ cognitive factor scores | 119 | 68 | 51 |
| Plasma NfL ~ cognitive factor scores | 155 | 91 | 64 |

## Table B.1 – B.2

|  | **F-value (df)** | **uncorr-p** | **FDR-p** |
| --- | --- | --- | --- |
| **CSF Biomarkers** |  |  |  |
| IL-1 β (log) | 1.034 (2, 160) | *0.358* | *0.716* |
| IL-6 (log) | 4.037 (2, 160) | *0.020 ^a^* | *0.120* |
| IL-8 (log) | 0.602 (2, 160) | *0.549* | *0.824* |
| IL-10 (log) | 0.352 (2, 160) | *0.704* | *0.845* |
| CRP (log) | 1.337 (2, 160) | *0.266* | *0.716* |
| TNF-α (log) | 0.079 (2, 160) | *0.924* | *0.924* |
| **Plasma Biomarkers** |  |  |  |
| IL-1 β (log) | 1.035 (2, 208) | *0.357* | *0.625* |
| IL-6 (log) | 1.804 (2, 208) | *0.167* | *0.390* |
| IL-8 (log) | 0.503 (2, 208) | *0.605* | *0.706* |
| IL-10 (log) | 0.715 (2, 208) | *0.491* | *0.687* |
| CRP (log) | 3.167 (2, 208) | *0.044 ^b^* | *0.154* |
| TNF-α (log) | 3.880 (2, 208) | *0.022 ^c^* | *0.154* |
| NfL (log) | 0.027 (2, 208) | *0.973* | *0.973* |

Results from Analyses of Covariance (ANCOVA) to assess differences in log-transformed cerebrospinal (CSF) and plasma biomarkers between three groups: (1) former American football players with NBD (Foot^NBD+^), (2) without NBD (Foot^NBD-^), and (3) unexposed individuals without NBD (UE^NBD-^). Models included covariates: age, BMI, rFSRP, and antidepressant use. Results are displayed with F-values, degrees of freedom (df), uncorrected p-values (uncorr-p), and FDR-correct p-values (FDR-p). Table B.2 displays group differences after stratification for age (Age<60 or Age≥60).

**Table B.1**

|  | **F-value (df)** | **uncorr-p** | **FDR-p** |
| --- | --- | --- | --- |
| **Age <60** |  |  |  |
| CSF IL-1 β (log) | 0.232 (2, 84) | *0.793* | *0.983* |
| CSF IL-6 (log) | 2.089 (2, 84) | *0.130* | *0.910* |
| CSF IL-8 (log) | 0.123 (2, 84) | *0.885* | *0.983* |
| CSF IL-10 (log) | 1.224 (2, 84) | *0.299* | *0.983* |
| CSF CRP (log) | 0.144 (2, 84) | *0.866* | *0.983* |
| CSF TNF-α (log) | 0.017 (2, 84) | *0.983* | *0.983* |
| Plasma NfL (log) | 0.043 (2, 116) | *0.958* | *0.983* |
| **Age ≥60** |  |  |  |
| CSF IL-1 β (log) | 1.017 (2, 69) | *0.367* | *0.556* |
| CSF IL-6 (log) | 2.384 (2, 69) | *0.100* | *0.303* |
| CSF IL-8 (log) | 0.698 (2, 69) | *0.501* | *0.585* |
| CSF IL-10 (log) | 1.053 (2, 69) | *0.354* | *0.556* |
| CSF CRP (log) | 2.101 (2, 69) | *0.130* | *0.303* |
| CSF TNF-α (log) | 0.070 (2, 69) | *0.792* | *0.792* |
| Plasma NfL (log) | 2.568 (2, 85) | *0.083* | *0.303* |

**Table B.2**

^a^ post hoc pair-wise comparisons: Foot^NBD+^ vs Foot^NBD-^ p=0.032; Foot^NBD+^ vs UE^NBD-^ p=0.071; Foot^NBD-^ vs UE^NBD-^ p=0.999

^b^ post hoc pair-wise comparisons: Foot^NBD+^ vs Foot^NBD-^ p=0.075; Foot^NBD+^ vs UE^NBD-^ p=0.954; Foot^NBD-^ vs UE^NBD-^ p=0.083

^c^ post hoc pair-wise comparisons: Foot^NBD+^ vs Foot^NBD-^ p=0.506; Foot^NBD+^ vs UE^NBD-^ p=0.135; Foot^NBD-^ vs UE^NBD-^ p=0.017

## Tables C.1 – C.5

Results from multivariable linear regression models between cerebrospinal (CSF) concentrations of Interleukin-1β, Interleukin-8, Interleukin-10, C-reactive protein (CRP) Tumor Necrosis Factor-alpha (TNF-α), (log-transformed) and neurobehavioral dysregulation (NBD) factor scores, within the entire cohort of former football players and stratified by age. Models included covariates: age, BMI, race, total AUDIT score, *APOE-ε4* carrier, revised Framingham Stroke Risk Profile score, and sleep apnea. Results are presented as standardized beta (β), with uncorrected and FDR-corrected p-values. * = p-values ≤0.05, ** = p-values ≤0.01.

| **CSF IL 1β (log)** | **β (95% CI)** | **uncorr-p** | **FDR-p** |
| --- | --- | --- | --- |
| **Entire cohort** | | | |
| Explosivity | 0.095 (-0.083 – 0.274) | 0.293 | 0.440 |
| Emotional Dyscontrol | 0.062 (-0.122 – 0.245) | 0.510 | 0.765 |
| Affective lability | 0.020 (-0.143 – 0.222) | 0.670 | 0.767 |
| Impulsivity | 0.018 (-0.162 – 0.198) | 0.844 | 0.868 |
| NBD total | 0.062 (-0.117 – 0.242) | 0.493 | 0.740 |
| **Age <60** | | | |
| Explosivity | 0.147 (-0.105 – 0.399) | 0.249 | 0.455 |
| Emotional Dyscontrol | 0.212 (-0.034 – 0.459) | 0.090 | 0.336 |
| Affective lability | 0.060 (-0.180 – 0.300) | 0.619 | 0.959 |
| Impulsivity | 0.135 (-0.114 – 0.385) | 0.283 | 0.679 |
| NBD total | 0.160 (-0.086 – 0.406) | 0.198 | 0.528 |
| **Age ≥60** | | | |
| Explosivity | -0.039 (-0.381 – 0.302) | 0.819 | 0.819 |
| Emotional Dyscontrol | -0.267 (-0.625– 0.091) | 0.140 | 0.336 |
| Affective lability | 0.028 (-0.347– 0.404) | 0.880 | 0.959 |
| Impulsivity | -0.336 (-0.672 – 0.001) | 0.051 | 0.153 |
| NBD total | -0.195 (-0.543 – 0.152) | 0.264 | 0.528 |

**Table C.1**

| **CSF IL-8 (log)** | **β (95% CI)** | **uncorr-p** | **FDR-p** |
| --- | --- | --- | --- |
| **Entire cohort** | | | |
| Explosivity | -0.007 (-0.182 – 0.196) | 0.941 | 0.941 |
| Emotional Dyscontrol | -0.008 (-0.202 – 0.186) | 0.933 | 0.933 |
| Affective lability | 0.045 (-0.158 – 0.228) | 0.720 | 0.767 |
| Impulsivity | -0.016 (-0.206 – 0.174) | 0.868 | 0.868 |
| NBD total | 0.006 (-0.184 – 0.195) | 0.953 | 0.953 |
| **Age <60** | | | |
| Explosivity | -0.070 (-0.328 – 0.188) | 0.590 | 0.644 |
| Emotional Dyscontrol | -0.033 (-0.289 – 0.223) | 0.800 | 0.862 |
| Affective lability | 0.006 (-0.238 – 0.250) | 0.959 | 0.959 |
| Impulsivity | -0.052 (-0.308 – 0.203) | 0.683 | 0.820 |
| NBD total | -0.040 (-0.293 – 0.212) | 0.752 | 0.752 |
| **Age ≥60** | | | |
| Explosivity | 0.154 (-0.153 – 0.460) | 0.318 | 0.455 |
| Emotional Dyscontrol | 0.029 (-0.304 – 0.362) | 0.862 | 0.862 |
| Affective lability | 0.131 (-0.207– 0.469) | 0.439 | 0.959 |
| Impulsivity | 0.028 (-0.291 – 0.347) | 0.860 | 0.860 |
| NBD total | 0.093 (-0.226 – 0.411) | 0.560 | 0.621 |

**Table C.2**

| **CSF IL-10** | **β (95% CI)** | **uncorr-p** | **FDR-p** |
| --- | --- | --- | --- |
| **Entire cohort** | | | |
| Explosivity | 0.148 (-0.028 – 0.323) | 0.098 | 0.293 |
| Emotional Dyscontrol | 0.094 (-0.087 – 0.275) | 0.306 | 0.612 |
| Affective lability | 0.075 (-0.106 – 0.255) | 0.413 | 0.767 |
| Impulsivity | 0.103 (-0.074 – 0.280) | 0.252 | 0.504 |
| NBD total | 0.120 (-0.057 – 0.296) | 0.182 | 0.364 |
| **Age <60** | | | |
| Explosivity | 0.120 (-0.123 – 0.364) | 0.328 | 0.455 |
| Emotional Dyscontrol | 0.162 (-0.078 – 0.402) | 0.183 | 0.366 |
| Affective lability | 0.080 (-0.151 – 0.311) | 0.491 | 0.959 |
| Impulsivity | 0.112 (-0.129 – 0.353) | 0.358 | 0.716 |
| NBD total | 0.138 (-0.100 – 0.375) | 0.252 | 0.528 |
| **Age ≥60** | | | |
| Explosivity | 0.229 (-0.040 – 0.498) | 0.093 | 0.455 |
| Emotional Dyscontrol | 0.045 (-0.254 – 0.344) | 0.762 | 0.862 |
| Affective lability | 0.132 (-0.172– 0.435) | 0.386 | 0.959 |
| Impulsivity | 0.067 (-0.219 – 0.353) | 0.638 | 0.820 |
| NBD total | 0.127 (-0.157 – 0.411) | 0.373 | 0.621 |

**Table C.3**

| **CSF TNF-α** | **β (95% CI)** | **uncorr-p** | **FDR-p** |
| --- | --- | --- | --- |
| **Entire cohort** | | | |
| Explosivity | 0.066 (-0.107 – 0.239) | 0.451 | 0.541 |
| Emotional Dyscontrol | -0.028 (-0.206 – 0.150) | 0.755 | 0.906 |
| Affective lability | -0.072 (-0.248 – 0.105) | 0.421 | 0.767 |
| Impulsivity | -0.054 (-0.228 – 0.119) | 0.536 | 0.804 |
| NBD total | -0.029 (-0.202 – 0.145) | 0.746 | 0.895 |
| **Age <60** |  |  |  |
| Explosivity | 0.215 (-0.038 – 0.468) | 0.094 | 0.455 |
| Emotional Dyscontrol | 0.115 (-0.139 – 0.370) | 0.368 | 0.631 |
| Affective lability | -0.044 (-0.288 – 0.199) | 0.717 | 0.959 |
| Impulsivity | -0.030 (-0.285 – 0.225) | 0.816 | 0.860 |
| NBD total | 0.072 (-0.180 – 0.324) | 0.569 | 0.621 |
| **Age ≥60** |  |  |  |
| Explosivity | -0.120 (-0.391 – 0.152) | 0.379 | 0.455 |
| Emotional Dyscontrol | -0.103 (-0.396 – 0.190) | 0.482 | 0.723 |
| Affective lability | -0.040 (-0.341– 0.261) | 0.789 | 0.959 |
| Impulsivity | 0.067 (-0.219 – 0.353) | 0.638 | 0.820 |
| NBD total | -0.088 (-0.369 – 0.193) | 0.532 | 0.621 |

**Table C.4**

| **CSF CRP (log)** | **β (95% CI)** | **uncorr-p** | **FDR-p** | |
| --- | --- | --- | --- | --- |
| **Entire cohort** | | | | |
| Explosivity | 0.118 (-0.061 – 0.297) | 0.195 | 0.390 | |
| Emotional Dyscontrol | 0.136 (-0.048 – 0.319) | 0.147 | 0.441 | |
| Affective lability | 0.028 (-0.157 – 0.212) | 0.767 | 0.767 | |
| Impulsivity | 0.153 (-0.027 – 0.332) | 0.094 | 0.282 | |
| NBD total | 0.124 (-0.056 – 0.304) | 0.174 | 0.364 | |
| **Age <60** | | | | |
| Explosivity | 0.186 (-0.087 – 0.458) | 0.178 | | 0.455 |
| Emotional Dyscontrol | 0.027 (-0.246– 0.301) | 0.842 | | 0.862 |
| Affective lability | 0.026 (-0.235 – 0.286) | 0.844 | | 0.959 |
| Impulsivity | 0.083 (-0.190 – 0.355) | 0.546 | | 0.820 |
| NBD total | 0.087 (-0.182 – 0.356) | 0.519 | | 0.621 |
| **Age ≥60** | | | | |
| Explosivity | 0.133 (-0.157 – 0.423) | 0.359 | | 0.455 |
| Emotional Dyscontrol | 0.410 (0.123 – 0.698) | 0.006** | | 0.072 |
| Affective lability | 0.101 (-0.219 – 0.422) | 0.526 | | 0.959 |
| Impulsivity | 0.431 (0.161 – 0.701) | 0.002** | | 0.017* |
| NBD total | 0.337 (0.053 – 0.620) | 0.021* | | 0.126 |

**Table C.5**

## Tables D.1 – D.2

Results from multivariable linear regression models between measures of level of exposure to repetitive head impacts; total years of football, and cumulative head impact index (CHII) linear acceleration, and log-transformed cerebrospinal (CSF) concentrations of inflammatory biomarkers; Interleukin-1β, Interleukin-8, Interleukin-10, C-reactive protein (CRP) Tumor Necrosis Factor-alpha (TNF-α). Models were performed within the entire cohort of former football players and age-stratified cohorts. Models included covariates: age, BMI, race, total AUDIT score, *APOE-ε4* carrier, revised Framingham Stroke Risk Profile score, and sleep apnea. Results are presented as standardized beta (β), with uncorrected and FDR-corrected p-values. * = p-values ≤0.05

| **Total years of football** | **β (95% CI)** | **uncorr-p** | **FDR-p** |
| --- | --- | --- | --- |
| **Entire cohort** | | | |
| CSF IL-1β | 0.042 (-0.135 – 0.218) | 0.639 | 0.760 |
| CSF IL-6 | 0.181 (0.002 – 0.360) | 0.048* | 0.144 |
| CSF IL-8 | 0.202 (0.039 – 0.365) | 0.016* | 0.095 |
| CSF IL-10 | 0.028 (-0.151 – 0.206) | 0.760 | 0.760 |
| CSF CRP | -0.070 (-0.244 – 0.105) | 0.431 | 0.760 |
| CSF TNF-α | 0.046 (-0.136 – 0.228) | 0.617 | 0.760 |
| **Age <60** | | | |
| CSF IL-1β | -0.122 (-0.363 – 0.119) | 0.315 | 0.498 |
| CSF IL-6 | 0.173 (-0.072– 0.418) | 0.163 | 0.406 |
| CSF IL-8 | 0.200 (-0.034 – 0.433) | 0.093 | 0.406 |
| CSF IL-10 | -0.011 (-0.261 – 0.240) | 0.931 | 0.963 |
| CSF CRP | -0.050 (-0.273 – 0.173) | 0.657 | 0.876 |
| CSF TNF-α | 0.006 (-0.236 – 0.247) | 0.963 | 0.963 |
| **Age ≥60** | | | |
| CSF IL-1β | 0.176 (-0.078 – 0.429) | 0.169 | 0.406 |
| CSF IL-6 | 0.238 (-0.061 – 0.536) | 0.116 | 0.406 |
| CSF IL-8 | 0.304 (0.034 – 0.575) | 0.028* | 0.336 |
| CSF IL-10 | 0.156 (-0.164 – 0.476) | 0.332 | 0.498 |
| CSF CRP | -0.026 (-0.329 – 0.277) | 0.864 | 0.963 |
| CSF TNF-α | 0.199 (-0.116 – 0.514) | 0.209 | 0.418 |

**Table D.1**

| **CHII linear acceleration** | **β (95% CI)** | **uncorr-p** | **FDR-p** |
| --- | --- | --- | --- |
| **Entire cohort** | | | |
| CSF IL-1β | -0.017 (-0.198 – 0.165) | 0.857 | 0.857 |
| CSF IL-6 | 0.148 (-0.037 – 0.333) | 0.117 | 0.240 |
| CSF IL-8 | 0.134 (-0.035 – 0.304) | 0.120 | 0.240 |
| CSF IL-10 | -0.150 (-0.331 – 0.031) | 0.104 | 0.240 |
| CSF CRP | -0.037 (-0.216 – 0.142) | 0.684 | 0.857 |
| CSF TNF-α | -0.032 (-0.219 – 0.155) | 0.735 | 0.857 |
| **Age <60** | | | |
| CSF IL-1β | -0.228 (-0.462 – 0.007) | 0.057 | 0.171 |
| CSF IL-6 | 0.065 (-0.181 – 0.312) | 0.598 | 0.797 |
| CSF IL-8 | 0.104 (-0.132 – 0.340) | 0.381 | 0.762 |
| CSF IL-10 | -0.259 (-0.499 – -0.019) | 0.035* | 0.156 |
| CSF CRP | -0.030 (-0.251 – 0.192) | 0.790 | 0.862 |
| CSF TNF-α | -0.106 (-0.344 – 0.131) | 0.375 | 0.762 |
| **Age ≥60** | | | |
| CSF IL-1β | 0.073 (-0.197 – 0.342) | 0.590 | 0.797 |
| CSF IL-6 | 0.363 (0.063 – 0.663) | 0.019* | 0.156 |
| CSF IL-8 | 0.300 (0.016 – 0.584) | 0.039* | 0.156 |
| CSF IL-10 | 0.001 (-0.336 – 0.339) | 0.993 | 0.993 |
| CSF CRP | -0.045 (-0.270 – 0.361) | 0.774 | 0.862 |
| CSF TNF-α | 0.102 (-0.230 – 0.435) | 0.538 | 0.797 |

**Table D.2**

## Table E.1

Results from sensitivity analyses, assessing the correlation between concentrations of each log-transformed plasma and CSF inflammatory biomarker. Pearson’s correlation coefficients (r) with 95% confidence intervals (CI) and FDR-corrected p-values are displayed.

| **Association between plasma and CSF concentrations** | **Correlation coefficient (r), (95% CI)** | **FDR-p** |
| --- | --- | --- |
| IL-1β (log) | 0.039 (-0.109 – 0.185) | 0.604 |
| IL-6 (log) | 0.020 (-0.128 – 0.166) | 0.795 |
| IL-8 (log) | 0.233 (0.089 – 0.368) | 0.002 |
| IL-10 (log) | 0.093 (-0.055 – 0.237) | 0.216 |
| CRP (log) | 0.729 (0.652 – 0.791) | <0.001 |
| TNF-α (log) | 0.059 (-0.089 – 0.204) | 0.435 |

## Table F.1

Results from multivariable linear regression models between plasma concentrations of Interleukin-1β, Interleukin-6, Interleukin-8, Interleukin-10, C-reactive protein (CRP) Tumor Necrosis Factor-alpha (TNF-α) (log-transformed) and neurobehavioral dysregulation (NBD) factor scores, within the entire cohort of former American football players. Models included covariates: age, BMI, race, total AUDIT score, *APOE-ε4* carrier, revised Framingham Stroke Risk Profile score, and sleep apnea. Results are presented as standardized beta (β), with uncorrected and FDR-corrected p-values.

|  | **β (95% CI)** | **uncorr-p** | **FDR-p** |
| --- | --- | --- | --- |
| **Plasma IL 1β (log)** |  |  |  |
| Explosivity | 0.022 (-0.132 – 0.176) | 0.778 | 0.821 |
| Emotional Dyscontrol | -0.006 (-0.161 – 0.150) | 0.944 | 0.944 |
| Affective lability | -0.013 (-0.174 – 0.147) | 0.870 | 0.870 |
| Impulsivity | 0.032 (-0.123 – 0.187) | 0.683 | 0.778 |
| NBD total | 0.008 (-0.146 – 0.162) | 0.917 | 0.980 |
| **Plasma IL-6 (log)** |  |  |  |
| Explosivity | -0.058 (-0.100 – 0.216) | 0.472 | 0.821 |
| Emotional Dyscontrol | -0.049 (-0.209 – 0.110) | 0.542 | 0.842 |
| Affective lability | 0.148 (-0.312 – 0.015) | 0.075 | 0.450 |
| Impulsivity | 0.055 (-0.104 – 0.215) | 0.494 | 0.765 |
| NBD total | -0.035 (-0.193 – 0.124) | 0.664 | 0.980 |
| **Plasma IL-8 (log)** |  |  |  |
| Explosivity | 0.018 (-0.140 – 0.176) | 0.821 | 0.821 |
| Emotional Dyscontrol | -0.047 (-0.206 – 0.112) | 0.561 | 0.842 |
| Affective lability | 0.025 (-0.140 – 0.189) | 0.770 | 0.870 |
| Impulsivity | 0.023 (-0.137 – 0.182) | 0.778 | 0.778 |
| NBD total | 0.002 (-0.156 – 0.160) | 0.980 | 0.980 |
| **Plasma IL-10 (log)** |  |  |  |
| Explosivity | 0.023 (-0.131 – 0.177) | 0.769 | 0.821 |
| Emotional Dyscontrol | -0.021 (-0.177 – 0.135) | 0.789 | 0.944 |
| Affective lability | -0.108 (-0.269 – 0.052) | 0.184 | 0.512 |
| Impulsivity | -0.052 (-0.208 – 0.103) | 0.510 | 0.765 |
| NBD total | -0.050 (-0.204 – 0.105) | 0.526 | 0.980 |
| **Plasma CRP (log)** |  |  |  |
| Explosivity | 0.084 (-0.076 – 0.243) | 0.302 | 0.821 |
| Emotional Dyscontrol | 0.052 (-0.109 – 0.213) | 0.525 | 0.842 |
| Affective lability | 0.049 (-0.117 – 0.216) | 0.560 | 0.840 |
| Impulsivity | 0.118 (-0.042 – 0.278) | 0.148 | 0.765 |
| NBD total | 0.087 (-0.072 – 0.247) | 0.281 | 0.980 |
| **Plasma TNF-α (log)** |  |  |  |
| Explosivity | -0.029 (-0.184 – 0.127) | 0.717 | 0.821 |
| Emotional Dyscontrol | 0.047 (-0.110 – 0.204) | 0.552 | 0.842 |
| Affective lability | 0.093 (-0.069 – 0.255) | 0.256 | 0.512 |
| Impulsivity | 0.063 (-0.093 – 0.220) | 0.426 | 0.765 |
| NBD total | 0.056 (-0.100 – 0.211) | 0.482 | 0.980 |

## Table G.1 – G.2

| **CSF IL-6 (log)** | **β (95% CI)** | **uncorr-p** | **FDR-p** |
| --- | --- | --- | --- |
| **Entire cohort** | | | |
| Emotional Dyscontrol | 0.226 (0.047 – 0.406) | 0.014* | 0.084 |
| Affective Lability | 0.258 (0.083 – 0.434) | 0.004** | 0.024* |
| Impulsivity | 0.289 (0.115 – 0.464) | 0.001*** | 0.006** |
| NBD total | 0.278 (0.104 – 0.451) | 0.002** | 0.012* |
| **Age ≥60** | | | |
| Impulsivity | 0.420 (0.141 – 0.699) | 0.004** | 0.024* |
| **CSF CRP (log)** | | | |
| **Age ≥60** | | | |
| Impulsivity | 0.447 (0.167 – 0.727) | 0.003** | 0.024* |
| **Plasma NfL (log)** | | | |
| **Age ≥60** | | | |
| Emotional Dyscontrol | 0.411 (0.144 – 0.679) | 0.003** | 0.015* |
| Impulsivity | 0.495 (0.253 – 0.738) | 0.0001*** | 0.001** |
| NBD total | 0.334 (0.071 – 0.597) | 0.014* | 0.047* |

Results from sensitivity analyses with cognition. Results are presented from multivariable linear regression models between fluid biomarkers and neurobehavioral dysregulation factor scores, which were significant in previous models. These models now included Verbal Memory Factor score (table D1) or Executive Functioning and Processing Speed (table D2), in addition to other covariates (age, BMI, race, total AUDIT score, *APOE-ε4* carrier, revised Framingham Stroke Risk Profile score, and sleep apnea). * = p-values ≤0.05, ** = p-values ≤0.01, *** = p-values ≤0.001

**Table G.1** adjusted for Verbal Memory

| **CSF IL 6 (log)** | **β (95% CI)** | **uncorr-p** | **FDR-p** |
| --- | --- | --- | --- |
| **Entire cohort** | | | |
| Emotional Dyscontrol | 0.226 (0.054 – 0.399) | 0.011* | 0.066 |
| Affective Lability | 0.277 (0.099 – 0.455) | 0.003** | 0.018* |
| Impulsivity | 0.286 (0.116 – 0.455) | 0.001*** | 0.006** |
| NBD total | 0.280 (0.110 – 0.449) | 0.001*** | 0.006** |
| **Age ≥60** | | | |
| Impulsivity | 0.402 (0.121 – 0.684) | 0.006** | 0.036* |
| **CSF CRP (log)** | | | |
| **Age ≥60** | | | |
| Impulsivity | 0.436 (0.164 – 0.709) | 0.002** | 0.024* |
| **Plasma NfL (log)** | | | |
| **Age ≥60** | | | |
| Emotional Dyscontrol | 0.334 (0.054 – 0.613) | 0.020* | 0.100 |
| Impulsivity | 0.484 (0.225 – 0.743) | 0.0004*** | 0.004** |
| NBD total | 0.289 (0.009 – 0.569) | 0.043* | 0.110 |

**Table G.2** adjusted for Executive Functioning and Processing Speed

## Tables H.1 – H.2

| **CSF IL-6 (log)** | **β (95% CI)** | **uncorr-p** | **FDR-p** |
| --- | --- | --- | --- |
| **Entire cohort** | | | |
| Emotional dyscontrol | 0.241 (0.065 – 0.417) | 0.008** | 0.048* |
| Affective lability | 0.277 (0.106 – 0.449) | 0.002** | 0.012* |
| Impulsivity | 0.300 (0.130 – 0.470) | 0.0007*** | 0.004** |
| NBD total | 0.288 (0.118 – 0.457) | 0.001** | 0.006** |
| **Age ≥60** | | | |
| Impulsivity | 0.424 (0.153 – 0.695) | 0.003** | 0.018* |
| **CSF CRP (log)** | | | |
| **Age ≥60** | | | |
| Impulsivity | 0.431 (0.150 – 0.711) | 0.004** | 0.018* |

Results from sensitivity analyses after excluding three individual cases with a medical history or current medication use that potentially affects the systemic immune system (E.1), or after including antidepressant use as additional covariate (E.2). Results are presented from multivariable linear regression models between fluid biomarkers and neurobehavioral dysregulation factor scores, which were significant in previous models. Models are corrected for: age, BMI, race, total AUDIT score, , *APOE-ε4* carrier, revised Framingham Stroke Risk Profile score, and sleep apnea. * = p-values ≤0.05, ** = p-values ≤0.01, *** = p-values ≤0.001

| **CSF IL-6 (log)** | **β (95% CI)** | **uncorr-p** | **FDR-p** |
| --- | --- | --- | --- |
| **Entire cohort** | | | |
| Emotional dyscontrol | 0.227 (0.051 – 0.403) | 0.012* | 0.072 |
| Affective lability | 0.285 (0.112 – 0.459) | 0.002** | 0.012* |
| Impulsivity | 0.288 (0.118 – 0.457) | 0.001** | 0.006** |
| NBD total | 0.281 (0.111 – 0.450) | 0.001** | 0.006** |
| **Age ≥60** | | | |
| Impulsivity | 0.272 (0.026 – 0.517) | 0.031* | 0.153 |
| **CSF CRP (log)** | | | |
| **Age ≥60** | | | |
| Impulsivity | 0.271 (0.020 – 0.522) | 0.035* | 0.153 |

**Table H.1** Adjusted for systemic inflammatory diseases and medication

**Table H.2** Adjusted for use of antidepressant(s)

# **References**

1. Patton JH, Stanford MS, Barratt ES. Factor structure of the Barratt impulsiveness scale. J Clin Psychol. 1995;51(6):768-74.

2. Baron IS. Behavior rating inventory of executive function. Child Neuropsychol. 2000;6(3):235-8.

3. Brown GL, Goodwin FK, Ballenger JC, Goyer PF, Major LF. Aggression in humans correlates with cerebrospinal fluid amine metabolites. Psychiatry Res. 1979;1(2):131-9.

4. Buss AH, Durkee A. An inventory for assessing different kinds of hostility. J Consult Psychol. 1957;21(4):343-9.

5. Moore SR, Gresham LS, Bromberg MB, Kasarkis EJ, Smith RA. A self report measure of affective lability. J Neurol Neurosurg Psychiatry. 1997;63(1):89-93.

6. Kaufer DI, Cummings JL, Ketchel P, Smith V, MacMillan A, Shelley T, et al. Validation of the NPI-Q, a brief clinical form of the Neuropsychiatric Inventory. J Neuropsychiatry Clin Neurosci. 2000;12(2):233-9.

7. Spielberger CD. STAXI-2: State-Trait Anger Expression Inventory-2 : Professional Manual: Psychological Assessment Resources;1999.

8. Katz DI, Bernick C, Dodick DW, Mez J, Mariani ML, Adler CH, et al. National Institute of Neurological Disorders and Stroke Consensus Diagnostic Criteria for Traumatic Encephalopathy Syndrome. Neurology. 2021;96(18):848-63.

9. Pulukuri SV, Fagle T, Trujillo-Rodriguez D, van Amerongen S, Bernick C, et al. Characterizing Neurobehavioral Dysregulation in Former American Football Players: Findings from the DIAGNOSE CTE Research Project. J Neuropsychiatry Clin Neurosci. Under review. 2024.

10. Banks SJ, Yhang E, Tripodis Y, Su Y, Protas H, Adler CH, et al. Clinical outcomes and tau pathology in retired football players: Associations with diagnosed and witnessed sleep apnea. Neurol Clin Pract. Revision under review. 2024.

11. Stern RA, White T. NAB, Neuropsychological Assessment Battery: Administration, scoring, and interpretation manual: Psychological Assessment Resources Lutz; 2003.

12. Reitan RM. Validity of the Trail Making Test as an indicator of organic brain damage. Perceptual and motor skills. 1958;8(3):271-6.

13. Smith A. Symbol digit modalities test: Western psychological services Los Angeles; 1973.
